# Nearctic *Dactylogyrus* species (Platyhelminthes, Monogenea) parasitizing cypriniform fishes in the context of morphology and phylogeny, with descriptions of seven new species[Fn FN1]

**DOI:** 10.1051/parasite/2023032

**Published:** 2023-08-17

**Authors:** Eva Řehulková, Mária Seifertová, Kateřina Francová, Andrea Šimková

**Affiliations:** Department of Botany and Zoology, Faculty of Science, Masaryk University Kotlářská 2 611 37 Brno Czech Republic

**Keywords:** Monogenea, *Dactylogyrus*, North America, Leuciscidae, Catostomidae, Morphology, Phylogeny

## Abstract

Ribosomal DNA sequences are currently available for 32 morphologically recognized species of *Dactylogyrus* parasitizing Nearctic cypriniforms, but only 16 of them are assigned to nominal species. Herein, morphological data on 28 of the 32 species are provided, together with comments on their phylogenetic relationships in the context of the morphology of taxonomically important structures. Seven new species of *Dactylogyrus* are described from five species of leuciscids and one species of catostomids, as follows: *D. aduncus* n. sp. from *Campostoma spadiceum*, *D. cloutmani* n. sp. from *Luxilus chrysocephalus isolepis*, *D. cornifrons* n. sp. from *Cyprinella venusta*, *D. fimbratus* n. sp. from *Rhinichthys cataractae*, *D. mcallisteri* n. sp. from *Semotilus atromaculatus*, and *D. chieni* n. sp. and *D. haneki* n. sp. from *Hypentelium nigricans*. Four previously described species of *Dactylogyrus*, *D. atromaculatus* from *S. atromaculatus*, *D. eos* from *C. neogaeus*, *D. parvicirrus* from *Notemigonus crysoleucas* and *D. perlus* from *L. c. isolepis*, are redescribed and/or figured. As for the remaining 17 species, only the male copulatory organs (MCOs) taken from the respective hologenophores are illustrated. On the basis of phylogenetic analyses, two main clades of Nearctic *Dactylogyrus* were recognized and supported by the different morphology of the MCO. The first one included 22 strictly Nearctic species sharing the same MCO type with *Dactylogyrus* spp. parasitizing cyprinids likely of Asian origin. The second clade encompassed *Dactylogyrus* spp. with diverse MCO morphology and was placed in the sister position to *Dactylogyrus* spp. parasitizing European leuciscids and North-West African cyprinids.

## Abstract (fr)

Des séquences d’ADN ribosomal sont actuellement disponibles pour 32 espèces morphologiquement reconnues de *Dactylogyrus* parasitant les cypriniformes néarctiques, mais seulement 16 d’entre elles sont attribuées à des espèces nominales. Ici, des données morphologiques sur 28 des 32 espèces sont fournies avec des commentaires sur leurs relations phylogénétiques dans le contexte de la morphologie des structures taxonomiquement importantes. Sept nouvelles espèces de *Dactylogyrus* sont décrites à partir de cinq espèces de leuciscidés et d’une espèce de catostomidés, comme suit : *D. aduncus* n. sp. de *Campostoma spadiceum*, *D. cloutmani* n. sp. de *Luxilus chrysocephalus isolepis*, *D. cornifrons* n. sp. de *Cyprinella venusta*, *D. fimbratus* n. sp. de *Rhinichthys cataractae*, *D. mcallisteri* n. sp. de *Semotilus atromaculatus* et *D. chieni* n. sp. et *D. haneki* n. sp. de *Hypentelium nigricans*. Quatre espèces de *Dactylogyrus* précédemment décrites, *D. atromaculatus* de *S. atromaculatus*, *D. eos* de *C. neogaeus*, *D. parvicirrus* de *Notemigonus crysoleucas* et *D. perlus* de *L. c. isolepis*, sont redécrites et/ou figurées. Comme pour les 17 espèces restantes, seuls les organes copulateurs mâles (OCM) prélevés sur les hologénophores respectifs sont illustrés. Sur la base d’analyses phylogénétiques, deux clades principaux de *Dactylogyrus* néarctiques ont été reconnus et soutenus par des morphologies différentes de l’OCM. Le premier comprenait 22 espèces strictement néarctiques partageant le même type d’OCM que les *Dactylogyrus* spp. parasitant des cyprinidés probablement d’origine asiatique. Le deuxième clade comprenait des *Dactylogyrus* spp. avec diverses morphologies d’OCM et était placé en position sœur des *Dactylogyrus* spp. parasitant les leuciscidés européens et les cyprinidés d’Afrique du Nord-Ouest.

## Introduction

The Cypriniformes are the most diverse clade of primary freshwater fishes [45], with more than 4700 valid species [85]. Recently, four main suborders (lineages) of this monophyletic group (*i.e.*, Catostomoidei, Cobitoidei, Cyprinoidei and Gyrinocheiloidei) have been proposed, with the Cyprinoidei containing multiple families including species previously classified in the Cyprinidae *sensu lato* [77, 83, 84]. The natural distribution range of the Cyprinoidei covers Eurasia, Africa and North America; however, out of a total of 12 families (including Cyprinidae *sensu stricto*), the Leuciscidae are the only one that is native to North America. The Catostomoidei (suckers) exhibit a Holarctic distribution, with their greatest diversity occurring in North America and only a single species present in Asia. The Cobitoidei (loaches) occur throughout Eurasia and northern Africa, and the Gyrinocheiloidei (algae eaters) are restricted to Southeast Asia [28]. Their great diversity and wide geographical distribution combined with our increasing knowledge of their phylogenetic relationships make cypriniforms attractive for studying ecological and evolutionary patterns and processes related to their closely associated parasites, such as highly host-specific monogeneans [3–5, 81, 82].

Monogeneans, a group of mainly ectoparasitic flatworms (Platyhelminthes) commonly found on the skin, fins, and gills of fishes, are among the most host-specific parasites (*e.g.*, [62, 87]). These flatworms, having monoxenous life cycles, are highly diverse both in terms of species richness (currently, 5522 species are known; [29]) and morphology (*i.e.*, they exhibit great morphological variety in the hard parts of the attachment and reproductive organs; [62]). Due to close evolutionary relationships with their fish hosts, monogeneans are generally considered useful biological tags for providing insights into their host’s taxonomy, phylogeny, and biogeography [7, 63]. There is no doubt that the basis for addressing ecological and evolutionary questions on parasites, including their host specificity and host-parasite coevolutionary interactions, is the correct identification of the studied species [27]. Parasite (as well as its host) misidentification, low taxonomic resolution (when, instead of species identification, only identification at the genus or family level is provided), and the occurrence of cryptic species are definite obstacles to resolving such questions [58, 64].

Cypriniforms are known to harbor gill-specific monogeneans of *Dactylogyrus* Diesing, 1850 (Dactylogyridae), one of the most speciose genera of helminths, with more than 900 nominal species [30]. The biogeography of *Dactylogyrus* spp. is undoubtedly closely related to the natural distribution of their cypriniform hosts, but records of these parasites vary widely in different regions of the world due to different levels of sampling efforts [30, 41]. Up to now, a total of 140 native species of cypriniforms have been reported as hosts of 224 species of monogeneans in North America [19, 20, 41, 47]. The majority of host species (*i.e.*, 110 spp.) belong to the Leuciscidae (Cyprinoidei) [20, 41], the largest freshwater fish clade in North America, including more than 310 species [28]. The remaining host records belong to the Catostomoidei, with 75 species currently recognized [1]. As *Dactylogyrus* is a highly diversified genus with almost exclusive host specificity to fishes of the Cyprinoidei [30], it is not surprising that more than half of the monogenean species (*i.e.*, 132 spp.; [19, 20, 41]) hitherto recorded on native North American cypriniforms belong to this genus.

The sclerotized structures of the attachment organ (*i.e.*, the haptor) and distal parts of the reproductive system [*i.e.*, the male copulatory organ (MCO) and vagina] play a major role in the description, delimitation, and discrimination of *Dactylogyrus* spp. However, many earlier taxonomic works on these parasites in North America are based on schematic illustrations of these taxonomically important structures, which probably gave rise to telescopic descriptions and poor differential diagnoses, resulting in many errors and several synonyms (*e.g.*, [18, 39, 52]).

In 2018, a survey was initiated to investigate the morphological and molecular diversity of monogeneans parasitizing native North American cypriniforms in order to obtain reliable data for an analysis of their phylogenetic relationships and evolutionary history. A total of 28 species of cypriniform fishes from four states in the United States (*i.e.*, Arkansas, Mississippi, New York, and Wisconsin) were examined. Besides other representatives of monogeneans, a total of 32 species of *Dactylogyrus* from the gills of 18 cypriniform host species were collected and morphologically recognized. Phylogenetic analyses based on concatenated 18S rDNA, ITS1, and 28S rDNA sequences showed that species of *Dactylogyrus* parasitizing Nearctic cypriniform fishes (16 species of Leuciscidae and two species of Catostomidae) form two independent clades with different origins [82]. The purpose of this study was to provide a connection between the molecular and morphological data on the specimens of *Dactylogyrus* spp. included in the phylogenetic analyses. In this paper, 28 of the 32 species of *Dactylogyrus* are morphologically vouchered together with illustrations of the MCOs taken from the respective hologenophores. Seven of the unidentified species of *Dactylogyrus* reported in Šimková *et al.* [82] are formally described here as new to science.

## Materials and methods

### Fish sampling

Fish hosts (two species of Catostomidae and 16 species of Leuciscidae) were captured by electrofishing or seine nets from several localities in four US states (Arkansas, Mississippi, New York, and Wisconsin) from 2018 to 2019 (Table 1). Fieldwork was carried out with the approval of the official local authorities (provided to US partners listed in the acknowledgements). Fishes were identified in the field by local collaborators familiar with the local fish fauna, and the identification was subsequently confirmed using sequences of the cytochrome b (cyt b) mitochondrial gene (see below). Scientific names and the systematic classification of fishes presented here follow Eschmeyer’s Catalog of Fishes [26]; host names used in the original descriptions of *Dactylogyrus* spp. are retained in parentheses as synonyms. Fishes were transported alive to the laboratory and kept in aerated containers until necropsied within three days of capture. They were killed by transection of the spinal cord and immediately examined for parasites.

**Table 1:** List of *Dactylogyrus* species used in phylogenetic analyses, their cypriniform host species, locality of collection, GenBank accession numbers for DNA sequences, and MNHN accession numbers for hologenophores.

*Dactylogyrus* species	Host species	Host family	Country	Body water (County)	GenBank No.		MNHN No.
					28S rDNA	18S rDNA+ITS1	Hologenophores
*D. aduncus* n. sp.	*Campostoma spadiceum*	Leuciscidae	Arkansas	Bear Creek (Garland Co.)	OM108544	OM108580	HEL 1954
*D. arcus* Rogers, 1967 [72]	*Luxilus chrysocephalus isolepis*	Leuciscidae	Arkansas*	Caddo River (Montgomery Co.)	OM108517	OM108553	HEL 1955
*D. atromaculatus* Mizelle, 1938 [51]	*Semotilus atromaculatus*	Leuciscidae	Wisconsin*	Baird Creek (Brown Co.)	OM108519	OM108555	HEL 1956-1957
*D. attenuatus* Mizelle & Klucka, 1953 [53]	*Semotilus atromaculatus*	Leuciscidae	Wisconsin	Baird Creek (Brown Co.)	OM108520	OM108556	HEL 1958
*D.* a*viunguis* Chien, 1974 [11]	*Nocomis biguttatus*	Leuciscidae	Wisconsin*	West Twin River (Brown Co.)	OM108521	OM108557	HEL 1959-1960
*D. bifurcatus* Mizelle, 1937 [50]	*Pimephales notatus*	Leuciscidae	Arkansas*	Big Fork Creek (Polk Co.)	OM108522	OM108558	HEL 1961
*D. boopsi* Cloutman, 1994 [17]	*Notropis telescopus* *	Leuciscidae	Arkansas	Big Fork Creek (Polk Co.)	OM108525	OM108561	HEL 1962
*D. bulbus* Mueller, 1938 [57]	*Luxilus chrysocephalus isolepis*	Leuciscidae	Arkansas*	Caddo River (Montgomery Co.)	OM108538	OM108574	HEL 1963
*D. cheloideus* Rogers, 1967 [72]	*Rhinichthys atratulus*	Leuciscidae	Wisconsin*	Baird Creek (Brown Co.)	OM108531	OM108567	HEL 1964
*D. chieni* n. sp.	*Hypentelium nigricans*	Catostomidae	Arkansas	Huddleston Creek (Montgomery Co.)	OM108545	OM108581	HEL 1965-1966
*D. chrosomi* Hanek *et al.* 1975 [32]	*Chrosomus neogaeus*	Leuciscidae	Wisconsin*	Mink River (Door Co.)	OM108526	OM108562	HEL1967-1968
*D. cloutmani* n. sp.	*Luxilus chrysocephalus isolepis*	Leuciscidae	Arkansas	Caddo River (Montgomery Co.)	OM108540	OM108576	HEL 1969-1970
*D. confusus* Mueller, 1938 [57]	*Clinostomus elongatus*	Leuciscidae	Wisconsin*	Baird Creek (Brown Co.)	OM108529	OM108565	HEL 1971
*D. cornifrons* n. sp.	*Cyprinella venusta*	Leuciscidae	Mississippi	Pascagoula River (Jackson Co.)	OM108542	OM108578	HEL 1972-1973
*D. cornu* Linstow, 1878 [66]	*Vimba vimba*	Leuciscidae	Czech Republic	–	KY629371	KY629342	–
*D. eos* Hanek *et al.* 1975 [32]	*Chrosomus neogaeus* *	Leuciscidae	Wisconsin*	Mink River (Door Co.)	OM108551	OM108587	HEL 1974
*D. ergensi* Molnár, 1964 [66]	*Chondrostoma vardarense*	Leuciscidae	Greece	–	MG792993	MG792878	–
*D. fimbratus* n. sp.	*Rhinichthys cataractae*	Leuciscidae	New York	Leatherstocking Creek (Otsego Co.)	OM108550	OM108586	HEL 1975
*D. flagristylus* Chien, 1974 [11]	*Nocomis biguttatus*	Leuciscidae	Wisconsin*	West Twin River (Brown Co.)	OM108530	OM108566	HEL 1976
*D. haneki* n. sp.	*Hypentelium nigricans*	Catostomidae	Arkansas	Huddleston Creek (Montgomery Co.)	OM108546	OM108582	HEL 1977-1978
*D. intermedius* Wegener, 1910 [66]**	*Carassius gibelio*	Cyprinidae	Czech Republic	–	OQ944102	OQ944103	–
*D. lachneri* Chien, 1971 [10]	*Nocomis biguttatus*	Leuciscidae	Wisconsin*	West Twin River (Brown Co.)	OM108532	OM108568	HEL 1979
*D. malleus* Linstow, 1877 [66]	*Barbus barbus*	Cyprinidae	Czech Republic	–	KY201112	KY201099	–
*D. marocanus* El Gharbi *et al.*, 1994 [66]**	*Carasobarbus fritschii*	Cyprinidae	Morocco	–	KY629355	KY629333	–
*D. mcallisteri* n. sp.	*Semotilus atromaculatus*	Leuciscidae	Arkansas	Big Fork Creek (Polk Co.)	OM108523	OM108559	HEL 1980-81-82
*D. nanus* Dogiel & Bychowsky, 1934 [66]	*Rutilus rutilus*	Leuciscidae	Czech Republic	–	AJ969942	AJ564145	–
*D. opsopoeodi* Rogers, 1967 [72]	*Opsopoeodus emiliae*	Leuciscidae	Mississippi*	Bluff Creek (Jackson Co.)	OM108533	OM108569	HEL 1983-1984
*D. ornatus* Rogers, 1967 [72]	*Notropis petersoni* *	Leuciscidae	Mississippi*	Bluff Creek (Jackson Co.)	OM108534	OM108570	HEL 1985-1986
*D. parvicirrus* Seamster, 1948 [78]	*Notemigonus crysoleucas*	Leuciscidae	New York*	Rom Hill Beaver Pond (Otsego Co.)	OM108527	OM108563	HEL 1987-1988
*D. pectenatus* Mayes, 1977 [46]	*Pimephales promelas*	Leuciscidae	Wisconsin*	Hickory Oak Pond (Door Co.)	OM108535	OM108571	HEL 1989
*D. perlus* Mueller, 1938 [57]	*Luxilus chrysocephalus isolepis*	Leuciscidae	Arkansas*	Caddo River (Montgomery Co.)	OM108536	OM108572	HEL 1990-1991
*D. rhinichthius* Wood & Mizelle, 1957 [88]	*Rhinichthys atratulus*	Leuciscidae	Wisconsin*	Baird Creek (Brown Co.)	OM1085371	OM108573	HEL 1992
*D. rutili* Gläser, 1965 [66]	*Leucos basak*	Leuciscidae	Albania	–	MG793012	MG792896	–
*D. scorpius* Rahmouni *et al.* 2017 [67]	*Luciobarbus rifensis*	Cyprinidae	Morocco	–	KX553860	KX578023	–
*D. simplexus* Monaco & Mizelle, 1955 [55]	*Pimephales notatus*	Leuciscidae	Arkansas*	Bear Creek (Garland Co.)	OM108528	OM108564	HEL 1993
*D. varius* Rahmouni *et al.* 2017 [67]	*Luciobarbus maghrebensi*s	Cyprinidae	Morocco	–	KX553863	KX578026	–
*D. vastator* Nybelin, 1924 [66]**	*Carassius gibelio*	Cyprinidae	Czech Republic	–	KY629366	KY201103	–
*D. venusti* Rogers, 1967 [72]	*Cyprinella venusta*	Leuciscidae	Mississippi *	Pascagoula River (Jackson Co.)	OM108552	OM108588	HEL 1994-1995

* New host or locality record.

** Species used as outgroup.

### Parasite sampling

Host gills were removed, placed in Petri dishes with tap water, and observed under a stereomicroscope (Olympus SZX7, Tokyo, Japan) for the presence of parasites. Monogeneans were collected using fine dissection needles and prepared according to Řehulková [70]. Some worms were mounted on slides under different levels of coverslip pressure and fixed with a mixture of glycerine and ammonium picrate (GAP) [44] for morphological analysis. Other specimens were bisected using fine needles: one half of the body (either the posterior part containing haptoral sclerites or the anterior part with the MCO) was fixed in 96% ethanol for later DNA extraction; the other half was mounted on a slide, fixed with GAP for species identification, and kept as a hologenophore (*sensu* Pleijel *et al.* [61]). The mounted specimens (or their parts) were studied using an Olympus BX61 (Tokyo, Japan) microscope equipped with phase contrast optics. Illustrations were made with the aid of a drawing attachment, scanned, and redrawn with a graphics tablet (Wacom Intuos5 Touch) compatible with Adobe Illustrator software (Adobe Systems Inc., San Jose, CA, USA). Measurements were captured using an Olympus digital camera and Stream Motion 1.9.2 image analysis software (Olympus). Measurements (in micrometers) are given as the mean followed by the range and the number (*n*) of specimens measured in parentheses. Body length includes the length of the haptor. The numbering of hook pairs follows the system recommended by Mizelle [49]. After morphometric analysis, the specimens (or their parts) fixed with GAP were re-mounted in Canada balsam following the procedure described by Ergens [25].

Type and voucher specimens of *Dactylogyrus* spp. were deposited in the Muséum National d’Histoire Naturelle (MNHN), Paris, France, as indicated in the respective species accounts and Table 1. For comparative purposes, the following type specimens of four species of *Dactylogyrus* deposited at the USNM collection were studied: *D. acicularis* Rogers, 1967 (USNM 061368; two paratypes), *D. apos* Mueller, 1938 (USNM 071443; three cotypes), *D. eos* Hanek, Molnár & Fernando, 1975 (USNM 73154; two paratypes), and *D. niger* Rogers & Mizelle, 1966 (USNM 060789; two paratypes).

### DNA extraction, amplification, and sequencing

For the molecular characterization of the studied species of *Dactylogyrus*, two rDNA fragments (a partial 28S rDNA and a fragment comprising a partial 18S rDNA and ITS1) were analyzed. The sequences of these target genes were generated as part of the previously published phylogenetic study [82], in which the DNA isolation, amplification, and sequencing processes were described in detail (see Table 1 for accession numbers).

Fish identification was confirmed using sequences of the cyt b mitochondrial gene and a sequence similarity approach employing the Basic Local Alignment Search Tool (https://blast.ncbi.nlm.nih.gov/Blast.cgi). Amplification of an approximately 1050 bp-long fragment of cyt b was performed using the primers GluF (forward, 5′–AACCACCGTTGTATTCAACTACAA–3′) and ThrR (reverse, 5′–ACCTCCGATCTTCGGATTACAAGACCG–3′) [43]. The DNA of host species was isolated from fin clips preserved in 96% ethanol using a DNeasy^®^ Blood & Tissue Kit (Qiagen, Hilden, Germany), following the manufacturer’s instructions. PCR reactions consisted of 1 U of Taq polymerase (Fermentas), 1× PCR buffer, 1.5 mM MgCl_2_, 0.4 mM of each dNTP, 0.4 μM of each primer, and an aliquot of 30 ng (1 μl) of genomic DNA in a total volume of 25 μL. The PCR was carried out in a Mastercycler ep gradient S instrument (Eppendorf) with the following steps: 2 min at 94 °C followed by 39 cycles of 45 s at 92 °C, 90 s at 48 °C, and 105 s at 72 °C, and 7 min of final elongation at 72 °C. PCR products were purified by ExoSAP–IT™ (Amplia, Bratislava, Slovakia) and were sequenced directly in both directions using the same primers as in the amplification reaction. Sequencing was carried out using a BigDye^®^ Terminator v3.1 Cycle Sequencing Kit (Applied Biosystems by Thermo Fisher Scientific, Prague, Czech Republic) and an Applied Biosystems 3130 Genetic Analyzer (Applied Biosystems). The obtained sequences were assembled and edited using Sequencer software (Gene Codes Corp., Ann Arbor, MI, USA).

### Phylogenetic analyses

Bayesian Inference (BI) and Maximum Likelihood (ML) analyses were performed on the concatenated dataset of 18S rDNA, ITS1 and 28S rDNA. The final nucleotide sequence alignment contained sequences of 28 North American *Dactylogyrus* species (7 new and 21 previously described) and seven species of *Dactylogyrus* from Palaearctic cyprinids and leuciscids representing members of phylogenetic *Dactylogyrus* lineage IV (see Šimková et al. [82]). Three *Dactylogyrus* species (*D. intermedius* Wegener, 1910, *D. vastator* Nybelin, 1937 and *D. marocanus* El Gharbi, Birgi & Lambert, 1994) representing members of phylogenetic *Dactylogyrus* lineage III (see Šimková *et al.* [82]) were used as an outgroup (Table 1). Sequences were aligned separately for each gene in MAFFT v.7 [37, 38] using the G-INS-i algorithm. The best-fit sequence substitution models were determined for each partition on the basis of the Bayesian Information Criterion (BIC) using ModelFinder [36] implemented in IQ-TREE [59]. The K2P+I+G model for 18S rDNA, the TIM2e+G model for ITS1, and the GTR+F+I+G model for 28S rDNA dataset were selected. BI analysis was conducted using MrBayes v.3.2 [76]. Two independent Metropolis-coupled Markov chain Monte Carlo (MCMCMC) analyses were run. Four chains were used (one heated, three cold), running for 10 million generations. Tree topologies were sampled every 100th generation, whereby the first 25% of trees from each run were discarded as burn-in. The remaining trees were used to construct majority-rule consensus trees and determine the Bayesian Posterior Probability (BPP) for each clade. The chain convergence and Effective Sampling Sizes (ESS) of all parameters were checked in Tracer v.1.7 [69]. ML analysis was performed in IQ-TREE v.2 [59] using a partition-based approach. To estimate the topological support, 1000 bootstrap replicates were calculated using UltraFast Bootstrap approximation (UFBoot) [34]. The obtained trees for BI and ML were visualized in FigTree v.1.4.3 [68].

## Results

Eighteen of the 28 species (64%) of North American cypriniforms examined for monogeneans were positive for *Dactylogyrus* spp. Two fish species, namely *Campostoma spadiceum* (Arkansas) and *Notropis petersoni* (Mississippi), were recorded as hosts of *Dactylogyrus* spp. as well as of monogeneans for the first time. Seven new and 21 previously described species of *Dactylogyrus* were described and reported; these species are listed in Table 1, including their host(s) and locality. Four species of *Dactylogyrus* (*i.e.*, *Dactylogyrus* sp. 3, 7, 8, and 9 reported in Šimková *et al.* [82], probably new to science, were not formally described here due to the insufficient number of specimens available. Twenty-eight *Dactylogyrus* species parasitizing mostly species of Nearctic Leuciscidae and forming two phylogenetic clades with different origins [82] are here divided into two main morphological groups on the basis of the MCO. Twenty-two species of strictly Nearctic *Dactylogyrus* form a monophyletic group and share the “nearctic” morphological type of MCO. This type is characterized by an accessory piece bifurcated into two unequal rami. To avoid confusion, we termed the individual rami “left” and “right” with respect to the medial body axis from the ventral view. The right ramus is usually shorter than the left one and possesses lightly sclerotized supplementary pieces supporting/guiding the copulatory tube. A link between the phylogenetic reconstruction and the morphological interspecific similarities of *Dactylogyrus* spp. presented in this study is discussed after the descriptions and redescriptions of the seven new and three previously described species, respectively.

### *Dactylogyrus aduncus* n. sp. (Fig. 1)

urn:lsid:zoobank.org:act:A88A82D5-3FC6-46D5-9A2E-CFDDF88827D3

**Figure 1:**
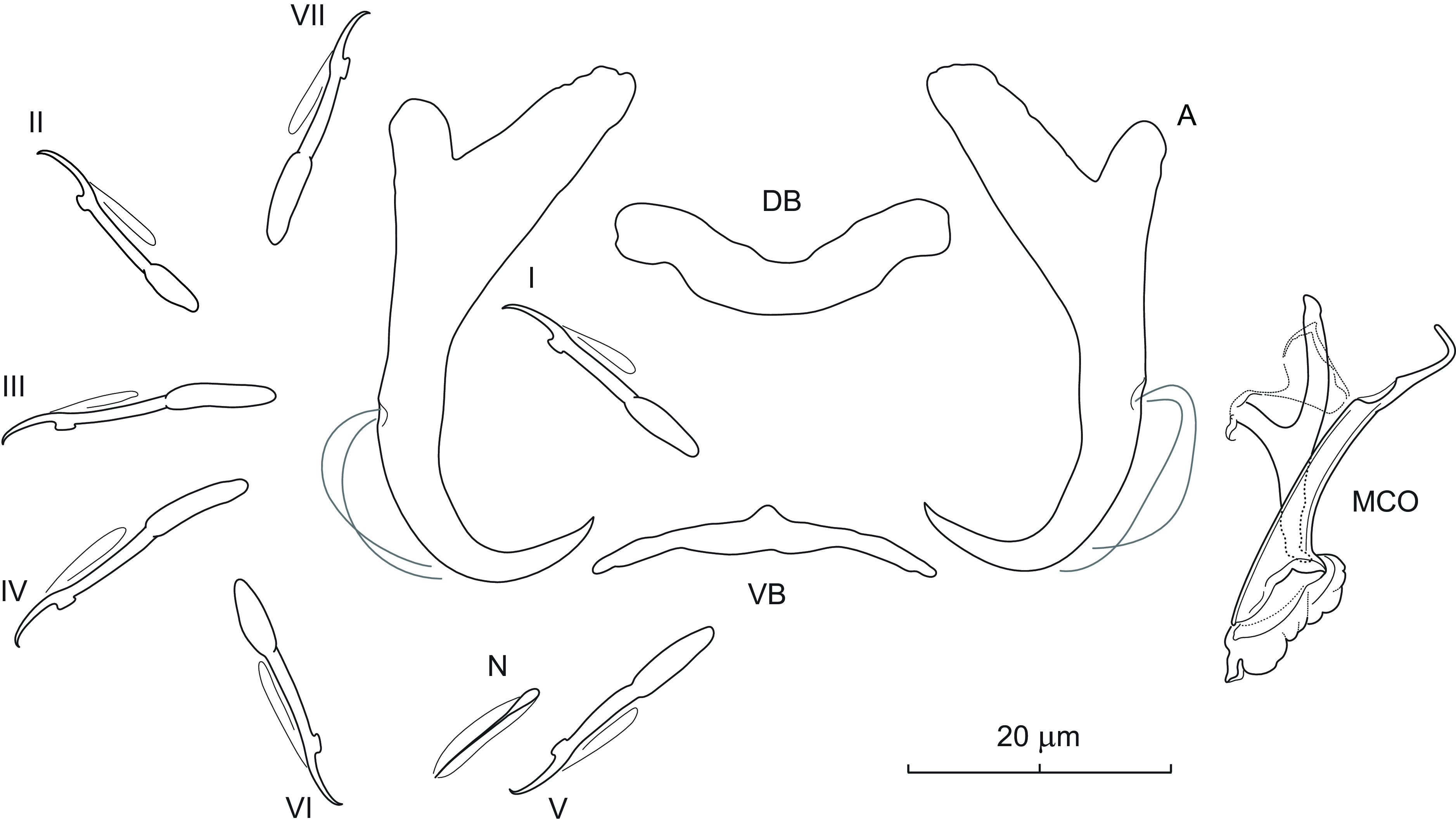
Sclerotized structures of *Dactylogyrus aduncus* n. sp. ex *Campostoma spadiceum*. A – anchor; DB – dorsal bar; VB – ventral bar; N – needle; I–VII – hooks; MCO – male copulatory organ.

Synonym: *Dactylogyrus* sp. 4 sensu Šimková et al. [82].

*Type host*: *Campostoma spadiceum* (Girard, 1856), Leuciscidae (Pogonichthyinae).

*Type locality*: Arkansas, Bear Creek (Garland Co.).

*Other locality*: Arkansas, Big Fork Creek (Polk Co.).

*Site on host*: Gill lamellae.

*Prevalence and intensity of infection*: 45% (5 fish infected/11 fish examined); 1–5 monogeneans per infected host.

*Type-specimens and specimens deposited*: Holotype (MNHN HEL1932); two paratypes (MNHN HEL1933-1934); one hologenophore (MNHN HEL1954).

*Etymology*: The specific name (an adjective) is from Latin (*aduncus* = hooked) and refers to the tip of the copulatory tube.

*Description* (based on 4 specimens in GAP and 6 hologenophores): Anchors thick, with moderately long inner root (approx. 2.5 times the length of outer root), well-developed rounded outer root, medially slightly constricted bent shaft, and point with recurved tip extending to level of tip of inner root. Dorsal bar yoke-shaped. Ventral bar broadly V-shaped with anteromedial knob. One pair of needles located near hooks of pair V. Hooks with delicate point, terminally flattened thumb, shank comprised of 2 subunits (proximal expansion 0.4–0.5 shank length); filamentous hooklet (FH) loop extending nearly to union of shaft subunits. MCO composed of basally articulated copulatory tube and accessory piece. Copulatory tube with funnel-shaped base surrounded by flange; shaft slightly bent, with subterminal opening and finger-like tip recurved at right angle. Accessory piece bifurcated into two unequal rami; right ramus shorter, with a delicate (poorly detectable even under phase contrast microscopy) sleeve-like membrane arising from its termination and appearing to be associated with the distal part of left ramus. Vagina not observed.

*Measurements*: Body 476 (469–484; *n* = 3) long; greatest width 68 (61–74; *n* = 3). Haptor 60 (59–60; *n* = 3) long, 98 (95–100; *n* = 3) wide. Anchor: total length 38 (34–40; *n* = 5); inner root length 14 (11–16; *n* = 5); outer root length 6 (5–7; *n* = 5); point length 10 (9–10; *n* = 5). Dorsal bar 26 (22–30; *n* = 5) long. Ventral bar 24 (20–29; *n* = 5) long. Hooks (I–VII) 19 (16–22; *n* = 5) long: pair I 18 (18–19), pair II 17 (16–18), pair III 21 (20–22), pair IV 22 (21–22), pair V 20 (19–22), pair VI 18 (17–19) and pair VII 19 (19–20). MCO: total straight length 30 (26–34; *n* = 5); tube trace length 26 (23–28; *n* = 5).

*Remarks*: *Dactylogyrus aduncus* n. sp. is easily differentiated from all other known congeners parasitizing North American cypriniforms by having a copulatory tube with a subterminal opening and finger-like termination recurved at a right angle. In addition, the anchors of the new species are relatively thick and possess a point with a recurved tip. This new species represents the first record of monogeneans on *C. spadiceum*. Up to now, four species of *Dactylogyrus* have been reported on two species of *Campostoma*, namely *D. acus* Mueller, 1938 (from *Campostoma anomalum* (Rafinesque); [57]), *D. georgiensis* Price, 1967 (from *C. anomalum*; [65]), *D. katherineae* Price, 1967 (from *C. anomalum* and *C. oligolepis* Hubbs & Green; [13, 14, 65]), and *D. semotilus* Wood & Mizelle, 1957 (from *C. anomalum*; [72]). Of the four species, *D. aduncus* n. sp. is most similar to *D. georgiensis*, from which it clearly differs, in addition to the above, by having a copulatory tube with comparatively smaller base.

### *Dactylogyrus cloutmani* n. sp. (Fig. 2)

urn:lsid:zoobank.org:act:F82C1D7D-B50B-453C-B30C-D398F7FCF0B8

**Figure 2:**
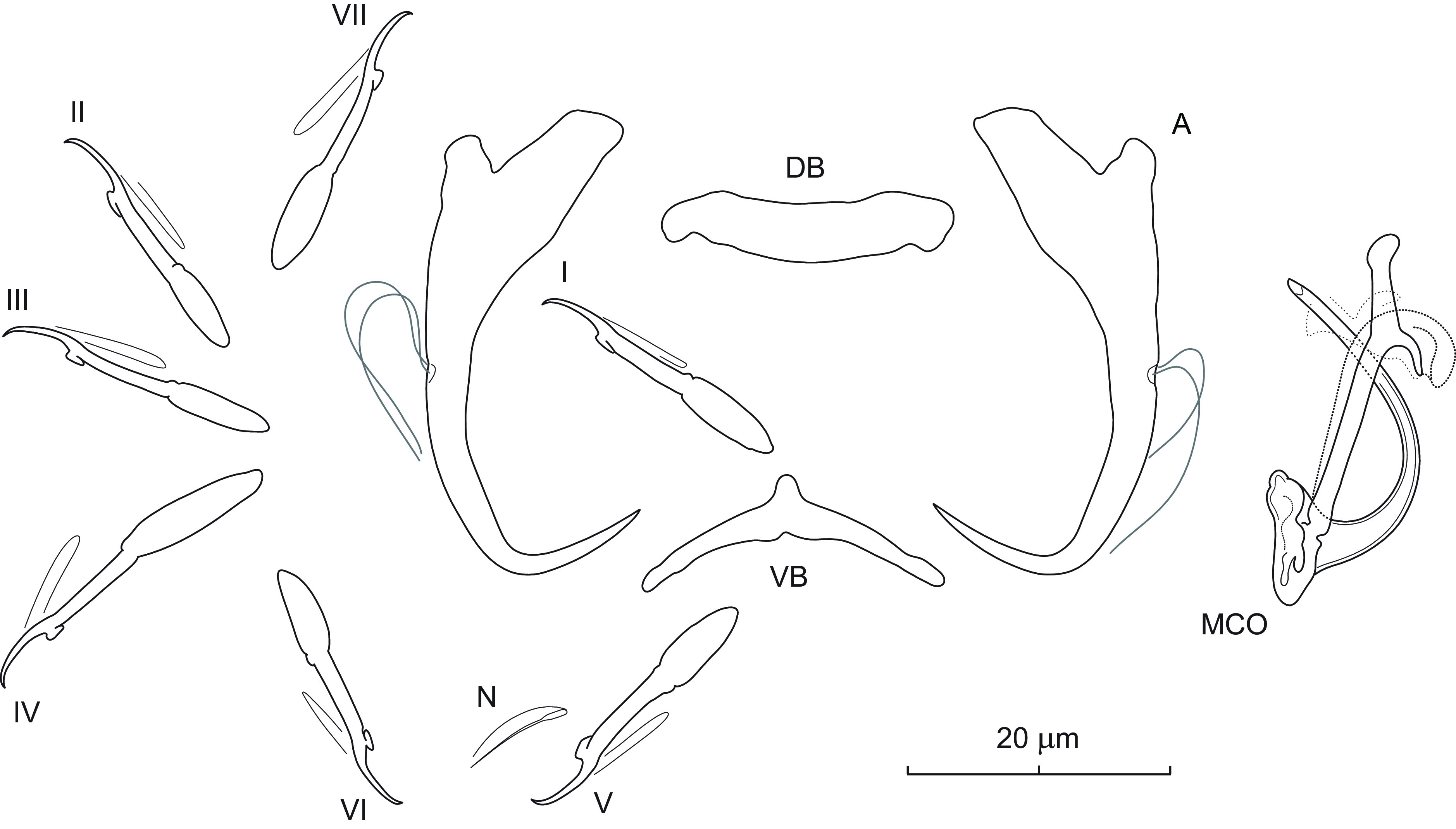
Sclerotized structures of *Dactylogyrus cloutmani* n. sp. ex *Luxilus chrysocephalus isolepis*. A – anchor; DB – dorsal bar; VB – ventral bar; N – needle; I–VII – hooks; MCO – male copulatory organ.

Synonym: *Dactylogyrus* sp. 1 sensu Šimková et al. [82].

*Type host*: *Luxilus chrysocephalus isolepis* (Hubbs & Brown, 1927), Leuciscidae (Pogonichthyinae).

*Type locality*: Arkansas, Caddo River (Montgomery Co.).

*Other locality*: Arkansas, Big Fork Creek (Polk Co.)

*Site on host*: Gill lamellae.

*Prevalence and intensity of infection*: 100% (3 fishes examined and infected); 3–8 monogeneans per infected host.

*Type-specimens and specimens deposited*: Holotype (MNHN HEL1939); six paratypes (MNHN HEL1940-1942); two hologenophores (MNHN HEL1969-1970).

*Etymology*: This species is named after Dr. Donald G. Cloutman (Kansas) in recognition of his contributions to the systematics and taxonomy of monogeneans parasitizing fishes in the United States.

*Description* (based on 10 specimens in GAP and 3 hologenophores): Anchors with moderately long inner root (approx. 2.5 times the length of outer root) having flattened termination, well-developed outer root, medially slightly constricted bent shaft, and straight recurved point slightly extending past level of tip of inner root. Dorsal bar nearly yoke-shaped. Ventral bar broadly V-shaped with anteromedial knob. One pair of needles located near hooks of pair V. Hooks with delicate point, small truncate thumb, shank comprised of 2 subunits (proximal expansion about 0.5 shank length); FH loop about 3/4 length of distal portion of shank. MCO composed of basally articulated copulatory tube and accessory piece. Copulatory tube with short funnel-shaped base; shaft arced, terminally tapering. Accessory piece bifurcated in its distal third into two unequal rami; left ramus longer, with rounded termination; right ramus shorter, thin, recurved posteriorly, merging with a weakly sclerotized ligament arising from the base of the accessory piece. Vagina not observed.

*Measurements*: Body 557 (493–731; *n* = 4) long; greatest width 86 (63–104; *n* = 4). Haptor 78 (70–84; *n* = 4) long, 102 (91–107; *n* = 4) wide. Anchor: total length 40 (35–41; *n* = 8); inner root length 11 (8–13; *n* = 8); outer root length 5 (3–7; *n* = 8); point length 10 (9–11; *n* = 8). Dorsal bar 23 (20–27; *n* = 8) long. Ventral bar 25 (24–27; *n* = 8) long. Hooks (I–VII) 24 (19–31; *n* = 8) long: pair I 23 (19–25) long, pair II 23 (20–25) long, pair III 28 (22–31), pair IV 24 (20–26), pair V 23 (20–26), pair VI 23 (19–25), and pair VII 25 (23–27). MCO: total straight length 29 (27–32; *n* = 8); tube trace length 31 (28–34; *n* = 8).

*Remarks*: *Dactylogyrus cloutmani* n. sp. is similar to *Dactylogyrus cursitans* Rogers, 1967 described from the same host species, *Luxilus chrysocephalus isolepis* (syn. *Notropis chrysocephalus isolepis*), in Alabama [72]. On the basis of Rogers’s original drawings of the sclerotized structures, *D. cloutmani* n. sp. differs from the latter species by the rounded termination of the left ramus (termination acute in *D. cursitans*) and by the presence of the ligament arising from the base of the accessory piece and merging with the right ramus into the form of a tongue-like grooved portion (probably serving as a guide for the distal third of the copulatory tube). In North America, *Luxilus chrysocephalus chrysocephalus* is among the cypriniform hosts with the highest monogenean species richness [41]. In the present study, specimens of *D. cloutmani* n. sp. co-occurred together with those of *Dactylogyrus arcus* Rogers, 1967, *Dactylogyrus bulbus* Mueller, 1938, and *Dactylogyrus perlus* Mueller, 1938. The description of *D. cloutmani* n. sp. increases the known number of *Dactylogyrus* spp. reported on this host species to 11.

### *Dactylogyrus cornifrons* n. sp. (Fig. 3)

urn:lsid:zoobank.org:act:5889A182-0F26-4A76-988D-6E5482474EE2

**Figure 3:**
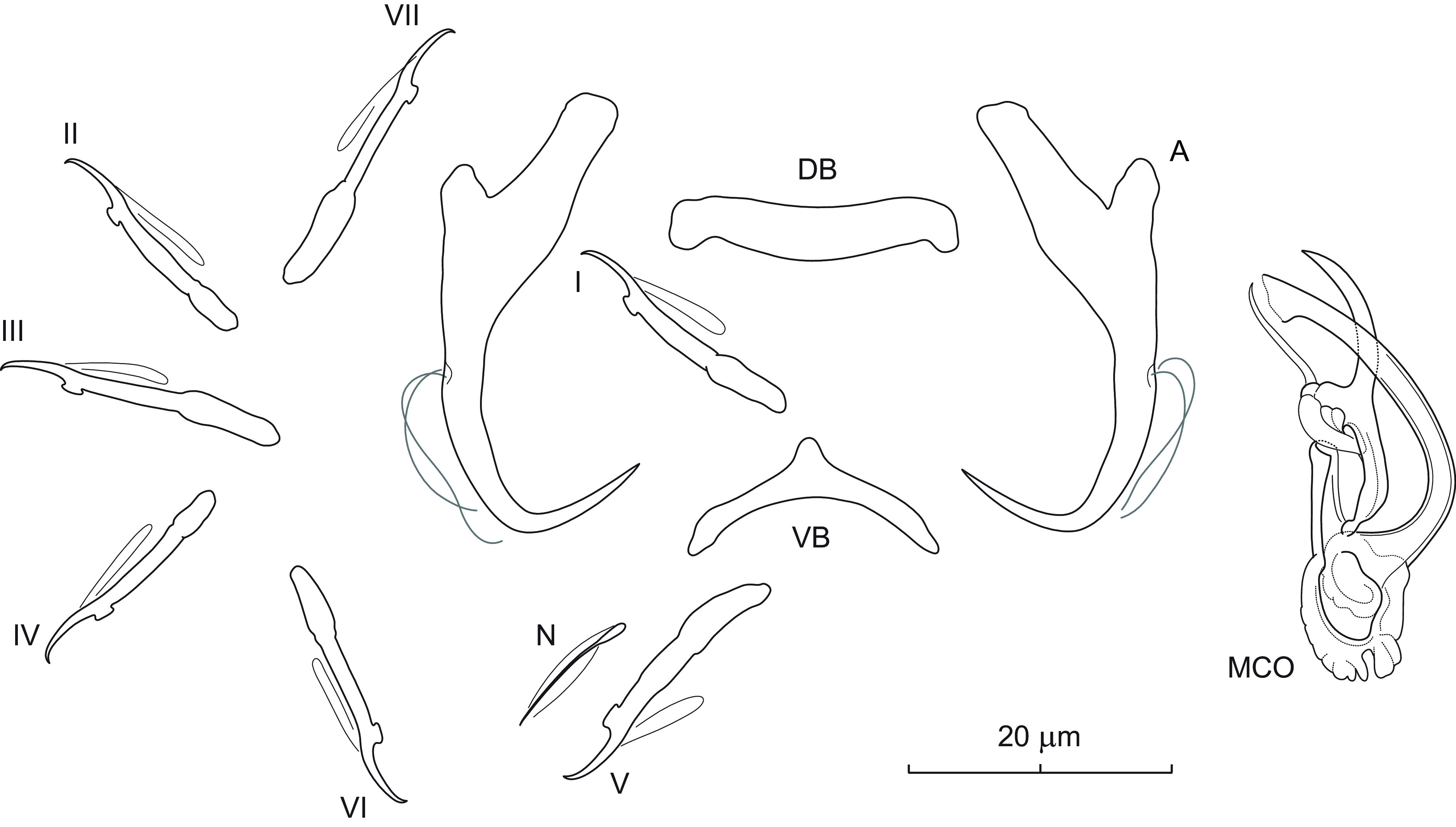
Sclerotized structures of *Dactylogyrus cornifrons* n. sp. ex *Cyprinella venusta*. A – anchor; DB – dorsal bar; VB – ventral bar; N – needle; I–VII – hooks; MCO – male copulatory organ.

Synonym: *Dactylogyrus* sp. 2 variant B sensu Šimková et al. [82].

*Type host*: *Cyprinella venusta* Girard, 1856, Leuciscidae (Pogonichthyinae).

*Type locality*: Mississippi, Pascagoula River (Moon Lake).

*Site on host*: Gill lamellae.

*Prevalence and intensity of infection*: 50% (6 of 12 fishes infected); 1–7 monogeneans per infected host.

*Type-specimens and specimens deposited*: Holotype (MNHN HEL1943); three paratypes (MNHN HEL1943-1945); two hologenophores (MNHN HEL1972-1973).

*Etymology*: The specific name (an adjective) is from Latin (*cornifrons* = having horns on the forehead) and refers to the shape of the accessory piece resembling the “sign of the horns” hand gesture.

*Description* (based on 6 specimens in GAP and 7 hologenophores): Anchors with moderately long inner root (approximately 2.5 times the length of outer root) having flattened termination, well-developed outer root, medially slightly constricted bent shaft, and straight recurved point slightly extending past level of tip of inner root. Dorsal bar nearly yoke-shaped. Ventral bar inverted V-shaped with anteromedial knob. One pair of needles located near hooks of pair V. Hooks with delicate point, small truncate thumb, shank comprised of 2 subunits (proximal expansion 0.3–0.4 shank length); FH loop about 3/4 length of distal portion of shank. MCO composed of basally articulated copulatory tube and accessory piece. Copulatory tube with base extended anteriorly into grooved heel-like projection; shaft nearly C-shaped, with flared distal end. Accessory piece medially bifurcated into two unequal rami; left ramus elongated, gently arched, slightly irregular in diameter, with diagonally truncate termination; right ramus terminally recurved into slightly sclerotized part forming a loop and bearing anteriorly projecting thin filament with acute termination. Vagina not observed.

*Measurements*: Body 410 (304–489; *n* = 3) long; greatest width 71 (52–92; *n* = 3). Haptor 55 (44–46; *n* = 3) long, 77 (68–86; *n* = 3) wide. Anchor: total length 31 (28–32; *n* = 5); inner root length 10 (9–12; *n* = 5); outer root length 4 (4–5; *n* = 5); point length 9 (8–9; *n* = 5). Dorsal bar 20 (17–22; *n* = 5) long. Ventral bar 18 (16–19; *n* = 5) long. Hooks (I–VII) 20 (16–23; *n* = 5) long: pair I 19 (17–20), pair II 19 (19–20), pair III 22 (20–23), pair IV 19 (18–20), pair V 20 (18–21), pair VI 18 (16–18), and pair VII 21 (20–22). MCO: total straight length 33 (29–38; *n* = 5); tube trace length 35 (30–41; *n* = 5).

*Remarks*: *Dactylogyrus cornifrons* n. sp. belongs to the group of congeners that have a base of the copulatory tube with an anteriorly protruding heel-like process (=heel). These include a number of *Dactylogyrus* species parasitizing North American Cyprinoidei, most frequently species of the Pogonichthyinae. The morphology of the haptoral sclerites and MCO in *D. cornifrons* n. sp. is most similar to that originally described for *Dactylogyrus venusti* Rogers, 1967 from the same host species in Alabama and Louisiana (see Figs. 182–188 in Rogers [72]). In the present study, both species co-occurred in the same host individuals and share the following characters: anchors with an elongate shaft and recurved straight point, a V-shaped ventral bar with an anteromedial knob-like projection, a thick nearly yoke-shaped ventral bar, a copulatory tube with a well-developed basal heel, and an accessory piece with the right ramus bearing an anteriorly projecting lightly sclerotized filament (see Fig. 12 for comparison). The left ramus and the filament curve inward to give them a pincer-like appearance. In young specimens of *D. cornifrons* n. sp., however, the filament is not well-developed in comparison to the other parts of the accessory piece, and therefore may not be detectable. *Dactylogyrus cornifrons* n. sp. clearly differs from *D. venusti* by having a copulatory tube with a shorter basal heel (*i.e.*, reaching the level of the proximal 1/3 of the shaft vs the level of the middle of the shaft in *D. venusti*) and an accessory piece with the right ramus terminally recurved into a slightly sclerotized loop (without a loop in *D. venusti*). In addition, in fixed specimens of *D. cornifrons* n. sp., the copulatory tube curves towards the right ramus (more specifically to its filament), while in *D. venusti* it is curved towards the left ramus, as in most North American *Dactylogyrus* spp. of the same (“nearctic”) type of MCO.

### *Dactylogyrus perlus* Mueller, 1938 (Fig. 4)

Synonym: *Dactylogyrus banghami* Mizelle & Donahue, 1944 [16].

**Figure 4:**
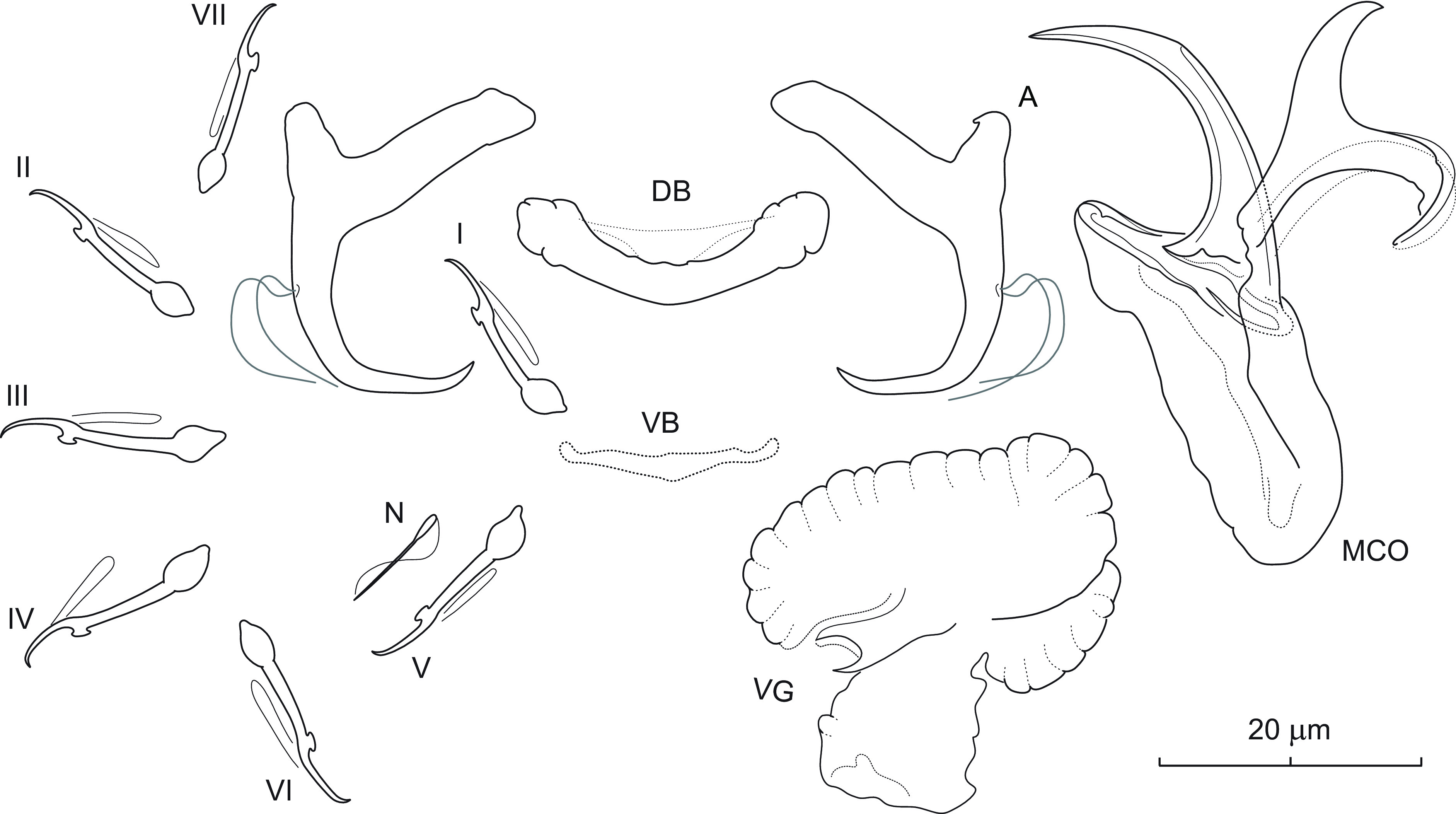
Sclerotized structures of *Dactylogyrus perlus* Mueller, 1938 ex *Luxilus chrysocephalus isolepis*. A – anchor; DB – dorsal bar; VB – ventral bar; N – needle; I–VII – hooks; VG – vagina; MCO – male copulatory organ.

*Type host*: *Luxilus cornutus* (Mitchill, 1817) (syn. *Notropis cornutus*), Leuciscidae (Pogonichthyinae).

*Type locality*: New York, Chautauqua Lake.

*Previous records*: *Luxilus cornutus*: New York [57], Ontario (as *D. banghami*; [52]). *Luxilus chrysocephalus*: Alabama (as *D. banghami* of Rogers [72], [18]), Tennessee [16].

*Current record*: *Luxilus chrysocephalus isolepis* (Hubbs & Brown, 1927): Arkansas, Big Fork Creek (Polk Co.) and Caddo River (Montgomery Co.).

*Site on host*: Gill lamellae.

*Prevalence and intensity of infection*: 83% (5 fish infected/6 fish examined); 3–10 monogeneans per infected host.

*Specimens deposited*: Three morphological vouchers together with *D. bulbus* (MNHN HEL1953); two hologenophores (MNHN HEL1990-1991).

*Redescription* (based on 8 specimens in GAP and 5 hologenophores): Anchor with markedly small base in comparison to size of roots; inner root long (approx. 3 times the length of outer root), nearly uniform in diameter, with flattened termination; outer root well-developed; shaft short, medially slightly constricted; point situated almost perpendicularly to shaft, with recurved tip, extending short of level of tip of inner root. Dorsal bar broadly V-shaped, with poorly visible medial membrane. Ventral bar vestigial, straight, with slight posteromedial expansion. One pair of needles located near hooks of pair V. Hooks with delicate point, small truncate thumb, shank comprised of 2 subunits (proximal expansion about 0.4 shank length); FH loop extending nearly to union of shaft subunits. MCO composed of basally articulated copulatory tube and accessory piece. Copulatory tube with robust base and short arched shaft; base extended anteriorly into relatively short and wide heel; shaft with distal 1/3 diagonally truncate. Accessory piece bifurcated medially into two unequal rami; left ramus with hooked termination; right ramus recurved posteriorly; a weakly sclerotized ligament arising from its base towards right ramus. Vagina a plate-like structure, appearing to be 3-lobed, with tunnel-like opening.

*Measurements*: Body 449 (289–631; *n* = 3) long; greatest width 82 (66–98; *n* = 3). Haptor 62 (40–82; *n* = 3) long, 83 (69–90; *n* = 3) wide. Anchor: total length 24 (22–25; *n* = 6); inner root length 13 (12–14; *n* = 6); outer root length 3 (3–5; *n* = 6); point length 8 (7–8; *n* = 6). Dorsal bar 22 (20–24; *n* = 6) long. Ventral bar 16 (15–18; *n* = 6) long. Hooks (I–VII) 15 (13–18; *n* = 6) long: pair I 15 (13–16), pair II 15 (14–17), pair III 16 (14–18), pair IV 15 (14–17), pair V 15 (14–16), pair VI 16 (14–17) and pair VII 15 (14–16). MCO: total straight length 43 (30–49; *n* = 6); tube trace length 30 (26–36; *n* = 6). Vagina 27 (20–39; *n* = 6) long, 25 (17–41; *n* = 6).

*Remarks*: *Dactylogyrus perlus* was found in association with three other species of *Dactylogyrus* (*i.e.*, *D. arcus*, *D. bulbus*, and *D. cloutmani* n. sp.) on the gills of the striped shiner collected in Arkansas (=new locality record). Unlike the three mentioned species, *D. perlus* belongs to the group of congeners that possess anchors with a point having a recurved tip and forming an acute or right angle with a relatively short shaft. The morphology of the haptoral and reproductive structures in the current specimens generally corresponds to that originally described by Mueller [57] and later redescribed by Mizelle and Donahue [52]. Our microscopic observation under phase-contrast optics additionally revealed the presence of an anteromedial membrane associated with the dorsal bar that is broadly V-shaped rather than straight, as depicted in the previous descriptions [52, 57].

After examination of the type specimens of *D. perlus* and *D. banghami* Mizelle & Donahue, 1944, Cloutman [16] considered the latter species as a junior synonym of *D. perlus*. Without providing any drawings and measurements, he based the synonymy on their mutual host and on the morphological similarity of the sclerotized structures in both species. Cloutman [16] stated that syntypes of *D. perlus* have an anteriorly directed process on the base of the copulatory tube (as in cotypes of *D. banghami*), a feature whose presence/absence Mizelle and Donahue [52] is reportedly considered important for distinguishing the two mentioned species. *Dactylogyrus banghami* has been reported from a variety of leuciscid host species and localities in eastern North America (see Hoffman [35]). However, according to Hanek *et al.* [32] and Cloutman [16], *D. banghami* (=*D. perlus*) represents a complex of morphologically similar species that needs to be revised, and in which *D. perlus* (*sensu stricto*) appears to be restricted on species of *Luxilus* Rafinesque [16, 18, 20]. The similarity of the species in the complex is given by the shape of both the anchors (see above in the remarks) and the MCO, which is composed of (i) a copulatory tube with a large base, having an anteriorly directed process (heel) and a slightly arched shaft with a diagonally truncated distal end, and (ii) an accessory piece medially bifurcated into two unequal rami (the left ramus with a hooked termination; the right ramus recurved posteriorly). Within the *D. perlus* (=*banghami*) complex, *D. perlus* (*s.s.*) most closely resembles *D. beckeri* Cloutman, 1987 and *D. confusus* Mueller, 1938 described from *Cyprinella galactura* (Cope) and *Clinostomus elongatus* (Kirtland) [15, 57], respectively, by having a relatively short basal heel in the copulatory tube. It differs from both species by having an accessory piece with rami of similar length (the left ramus is slightly longer than the right one in *D. beckeri* and *D. confusus*). In addition, *D. perlus* differs from *D. beckeri* by having a smaller MCO (*i.e.*, 43 (30–49) *vs* 58 (46–65) in *D. beckeri*), while the anchors are of similar size in both species, and from *D. confusus* by having a basal heel of the copulatory tube shorter than the remaining part of the base (measured from the midpoint of the base opening) (the basal heel is slightly longer in *D. confusus*).

### *Dactylogyrus mcallisteri* n. sp. (Fig. 5)

urn:lsid:zoobank.org:act:583F022F-16CB-49C0-A3F7-313B9203DBB1

**Figure 5:**
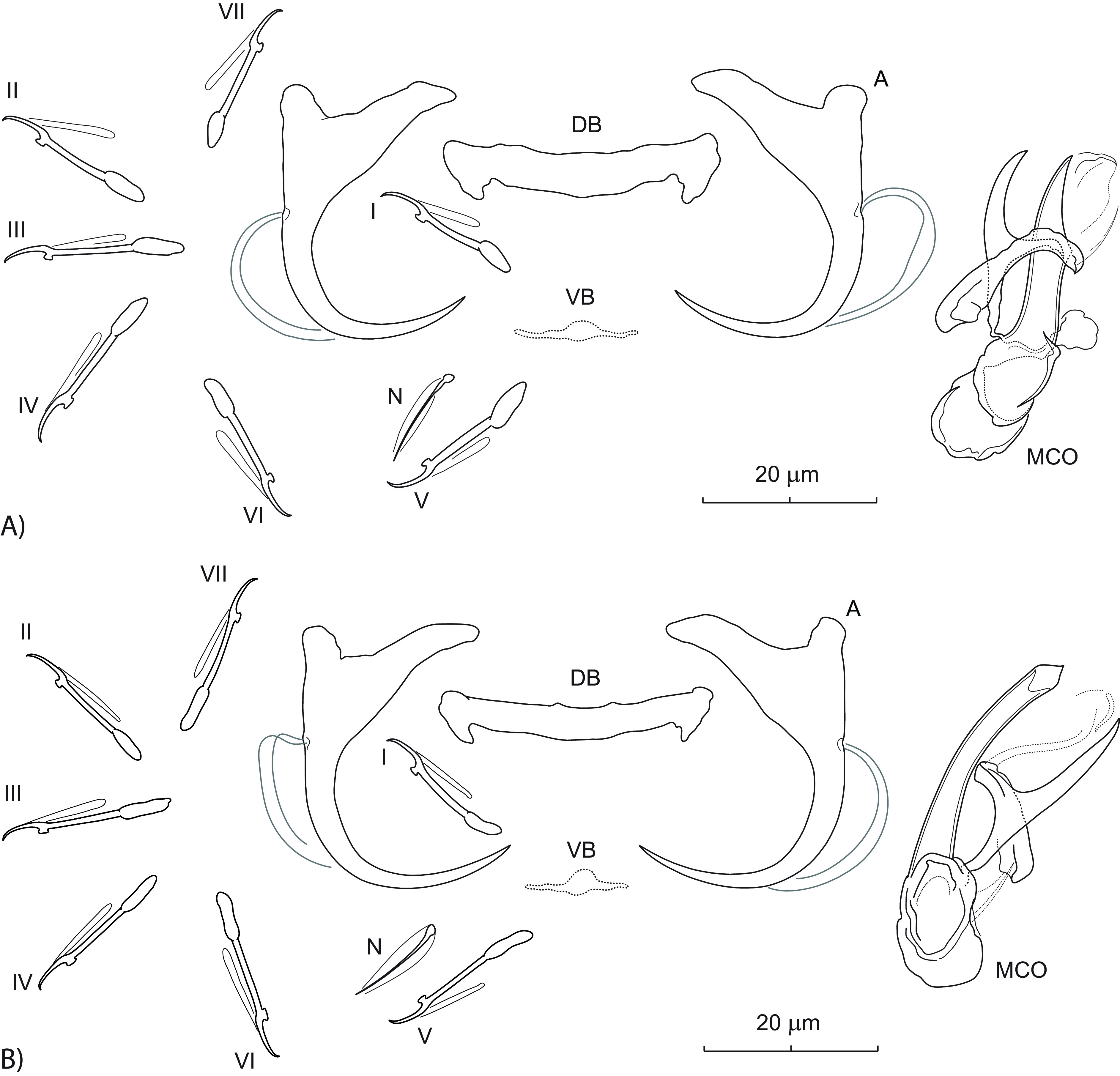
Sclerotized structures of *Dactylogyrus mcallisteri* n. sp. (A) and *D. atromaculatus* Mizelle, 1938 (B) ex *Semotilus atromaculatus* from Arkansas and Wisconsin, respectively. A – anchor; DB – dorsal bar; VB – ventral bar; N – needle; I–VII – hooks; MCO – male copulatory organ.

Synonym: *Dactylogyrus cf. atromaculatus* variant A sensu Šimková et al. [82].

*Type host*: *Semotilus atromaculatus* (Mitchill, 1818), Leuciscidae (Plagopterinae).

*Type locality*: Arkansas, Big Fork Creek (Polk Co.).

*Other locality*: Arkansas, Bear Creek (Garland Co.).

*Site on host*: Gill lamellae.

*Prevalence and intensity of infection*: 100% (7 fish infected/7 fish examined); 3–15 monogeneans per infected host.

*Type-specimens and specimens deposited*: Holotype (MNHN HEL1949); three hologenophores (MNHN HEL1980-1982).

*Etymology*: This species is named after Dr. Chris T. McAllister, Eastern Oklahoma State College, Idabel, Oklahoma, in recognition of his contributions to the taxonomy and systematics of North American fish parasites.

*Description* (based on 5 specimens in GAP and 3 hologenophores): Anchors with moderately long inner root (approx. 3 times the length of outer root) having flattened and slightly recurved termination, well-developed outer root, evenly curved shaft and point; point elongated, extending past level of tip of inner root. Dorsal bar rod shaped, with ends bevelled inwards. Ventral bar vestigial, with anteromedial bulbous expansion. One pair of needles located near hooks of pair V. Hooks with delicate point, small truncate thumb, shank comprised of 2 subunits (proximal expansion approx. 0.4 shank length); FH loop about 3/4 length of distal portion of shank. MCO composed of basally articulated copulatory tube and accessory piece. Copulatory tube with base having anterior and posterior flange; shaft relatively short (slightly longer than base), straight to slightly bent, with distal smaller half diagonally truncate. Accessory piece medially bifurcated into two unequal rami; left ramus elongated, gently arched, almost corresponding in shape and size to the distal half of the tube; right ramus bearing a plate passing diagonally across the base of the accessory piece.

*Measurements*: Body 667 (593–755; *n* = 3) long; greatest width 94 (75–115; *n* = 3). Haptor 66 (63–71; *n* = 3) long, 121 (103–139; *n* = 3) wide. Anchor: total length 32 (30–33; *n* = 5); inner root length 14 (13–15; *n* = 5); outer root length 4 (3–5; *n* = 5); point length 14 (13–14; *n* = 5). Dorsal bar 31 (29–33; *n* = 5) long. Ventral bar 14 (13–15; *n* = 5) long. Hooks (I–VII) 19 (17–22; *n* = 3) long: pair I 18 (17–19), pair II 19 (17–20), pair III 21 (20–22), pair IV 21 (20–22), pair V 20 (19–21), pair VI 19 (18–20), and pair VII 18 (18–19). MCO: total straight length 36 (33–38; *n* = 5); tube trace length 36 (34–38; *n* = 5).

*Remarks*: *Dactylogyrus mcallisteri* n. sp. is morphologically and genetically close to *Dactylogyrus atromaculatus* Mizelle, 1938, which was described and recorded on the same host species in Illinois [51], Ontario [24], and Wisconsin (present study). Both species possess almost identical haptoral structures (see Fig. 5), but they are easily distinguished by the comparative morphology of the MCO: (i) the basal flange of the copulatory tube extends anteriorly into a cloud shaped structure in *D. mcallisteri* n. sp. (absent in *D. atromaculatus*); (ii) the copulatory tube of *D. mcallisteri* n. sp. is characterized by a shaft almost the same length as its base (the shaft is thinner and longer in *D. atromaculatus*); and (iii) the distal diagonally truncated (or attenuated) part of the shaft goes almost one time into the shaft length in the new species (vs *ca.* 4 times into the shaft length in *D. atromaculatus*). Within North American cypriniform fishes, *Semotilus atromaculatus* is known as the host with the second-highest species richness of monogeneans [41]. The finding of *D. mcallisteri* n. sp. raises the species numbers of monogeneans and *Dactylogyrus* known from this host species to thirteen and nine, respectively.

### *Dactylogyrus chieni* n. sp. (Fig. 6)

urn:lsid:zoobank.org:act:BF65A554-E6D2-40B2-9764-49C8C4721070

**Figure 6:**
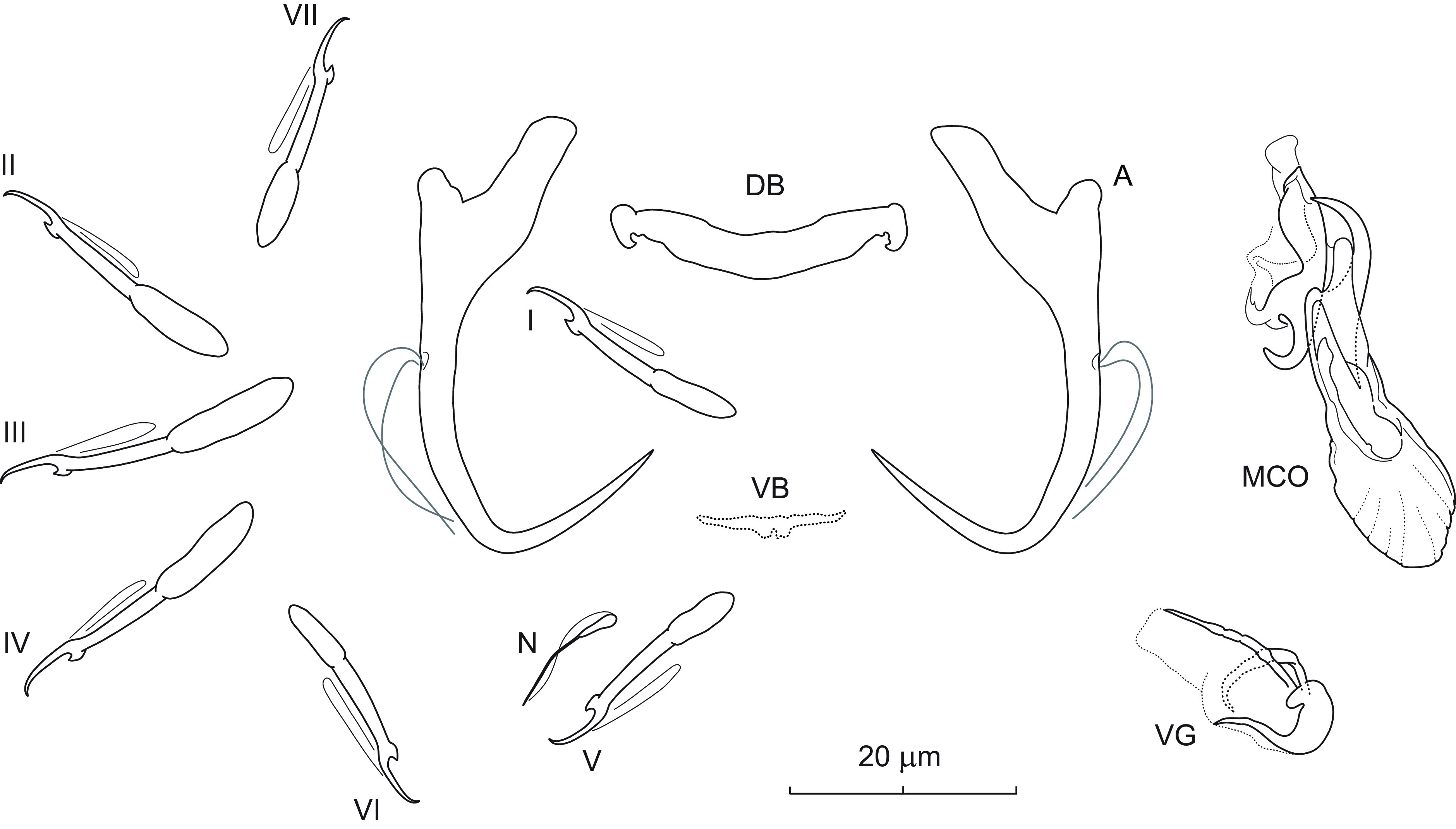
Sclerotized structures of *Dactylogyrus chieni* n. sp. ex *Hypentelium nigricans*. A – anchor; DB – dorsal bar; VB – ventral bar; N – needle; I–VII – hooks; VG – vagina; MCO – male copulatory organ.

Synonym: *Dactylogyrus* sp. 5 of Šimková et al. [82].

*Type host*: *Hypentelium nigricans* (Lesueur, 1817), Catostomidae (Catostominae).

*Type locality*: Arkansas, Huddleston Creek (Montgomery Co.).

*Other locality*: Arkansas, Walnut Creek (Garland Co.).

*Site on host*: Gill lamellae.

*Prevalence and intensity of infection*: 100% (3 fish infected/3 fish examined); 2–5 monogeneans per infected host.

*Type-specimens and specimens deposited*: Holotype (MNHN HEL1935); five paratypes (MNHN HEL1936-1938); two hologenophores (MNHN HEL1965-1966).

*Etymology*: This species is named after Dr. Shih Ming Chien in recognition of his contributions to the systematics and taxonomy of monogeneans parasitizing fishes in the United States.

*Comparative material studied*: *D. apos* Mueller, 1938 (USNM 071443; three cotypes) from *H. nigricans*; *D. niger* Rogers & Mizelle, 1966 (USNM 060789; two paratypes) from *Moxostoma duquesnei*.

*Description* (based on 9 specimens in GAP and 3 hologenophores): Anchors with elongate inner root (approx. 3 times the length of outer root) having slightly flattened termination, well-developed outer root, medially slightly constricted bent shaft, and straight sharply recurved point extending past level of tip of inner root. Dorsal bar rod-shaped or gently bent posteriorly in midregion, with subterminal bilateral notches on posterior border. Ventral bar vestigial, rod-shaped, with slight posteromedial expansion and irregular margins. One pair of needles located near hooks of pair V. Hooks with delicate point, upright acute thumb, shank comprised of 2 subunits (proximal expansion 0.4–0.5 shank length); FH loop extending nearly to union of shaft subunits. MCO robust, composed of basally articulated copulatory tube and accessory piece. Copulatory tube with markedly elongate base and short arched shaft; base extended anteriorly into poorly defined heel-like projection. Accessory piece broadly V-shaped, with virtually non-existent base, appearing articulated to the base of copulatory tube at its midlength; left (posterior) ramus distally with two claws in tandem position (subterminal claw with serrated margins, appearing lightly sclerotized and bearing poorly defined membrane); right (anterior) ramus grooved, distally with lightly sclerotized rounded extension. Vagina scoop-shaped, with three prominent spikes; one spike curved; two (long, short) relatively straight.

*Measurements*: Body 343 (253–417; *n* = 3) long; greatest width 50 (43–60; *n* = 3). Haptor 52 (47–55; *n* = 3) long, 62 (59–69; *n* = 3) wide. Anchor: total length 34 (32–38; *n* = 9); inner root length 11 (9–13; *n* = 9); outer root length 3 (3–4; *n* = 9); point length 13 (11–15; *n* = 9). Dorsal bar 23 (21–26; *n* = 9) long. Ventral bar 12 (10–13; *n* = 9) long. Hooks (I–VII) 22 (18–29; *n* = 6) long: pair I 21 (19–23), pair II 23 (21–27), pair III 26 (24–29), pair IV 25 (23–28), pair V 20 (19–22), pair VI 21 (18–23) and pair VII 20 (19–22). MCO: total straight length 31 (29–34; *n* = 9); tube trace length 24 (22–27; *n* = 9). Vagina: greatest length 16 (10–23; *n* = 9); greatest width 7 (6–10; *n* = 9).

*Remarks*: *Dactylogyrus chieni* n. sp. belongs to the group of congeners having an MCO with a huge elongate base, short arched shaft, and a broadly V-shaped accessory piece. These include *D. acicularis* Rogers, 1967, *D. apos* Mueller, 1938, *D. duquesni* Mueller, 1938, *D. hamatus* Rogers & Mizelle, 1966, *D. haneki* n. sp., *D. niger* Rogers & Mizelle, 1966 and *D. plumbeus* Rogers & Mizelle, 1966, all parasites of moxostomatins and thoburniins (Catostominae). In addition, all of these species share morphologically similar haptoral structures: anchors with an elongate shaft (*i.e.*, longer than the part including the base and roots) and recurved straight point, a relatively straight dorsal bar, and a delicate (vestigial) ventral bar with a slight medial expansion and irregular margins. *Dactylogyrus chieni* n. sp. seems to be different from all the species in the group by possessing a heel-like projection protruding anteriorly from the distal part of the base of the copulatory tube. However, this feature was not very visible in some specimens examined, and therefore its presence/absence in the species of the group could not be verified with certainty without a deeper examination of their type specimens. Leaving aside this feature, *D. chieni* n. sp. appears most similar to *D. niger* (from *Moxostoma duquesnei* (Lesueur), Alabama; [73]) by having a relatively stout accessory piece with a virtually non-existent base and rami of similar length that are positioned laterally and nearly parallel to the shaft of the copulatory tube. *Dactylogyrus chieni* n. sp. clearly differs from *D. niger* by having the left (posterior) ramus distally modified into two claws in a tandem position (the posterior ramus appearing simple and having a slightly hooked termination in *D. niger*). According to this character, *D. chieni* n. sp. seems to be similar to *D. apos* described from the same host species in New York by Mueller [57]. However, Mueller’s [57] three original drawings of the MCO clearly show the accessory piece as having a short base (*vs* a non-visible base in *D. chieni* n. sp.) bifurcated into two rami which lie perpendicular to the shaft of the copulatory tube. In addition, the right ramus of the accessory piece in *D. apos* is curved terminally around the distal part of the copulatory tube, while the respective ramus in *D. chieni* n. sp. is grooved and distally extended with a lightly sclerotized rounded flap.

### *Dactylogyrus haneki* n. sp. (Fig. 7)

urn:lsid:zoobank.org:act:087E6D6F-CB20-44D2-A1D8-A9FA0F09A4CF

**Figure 7:**
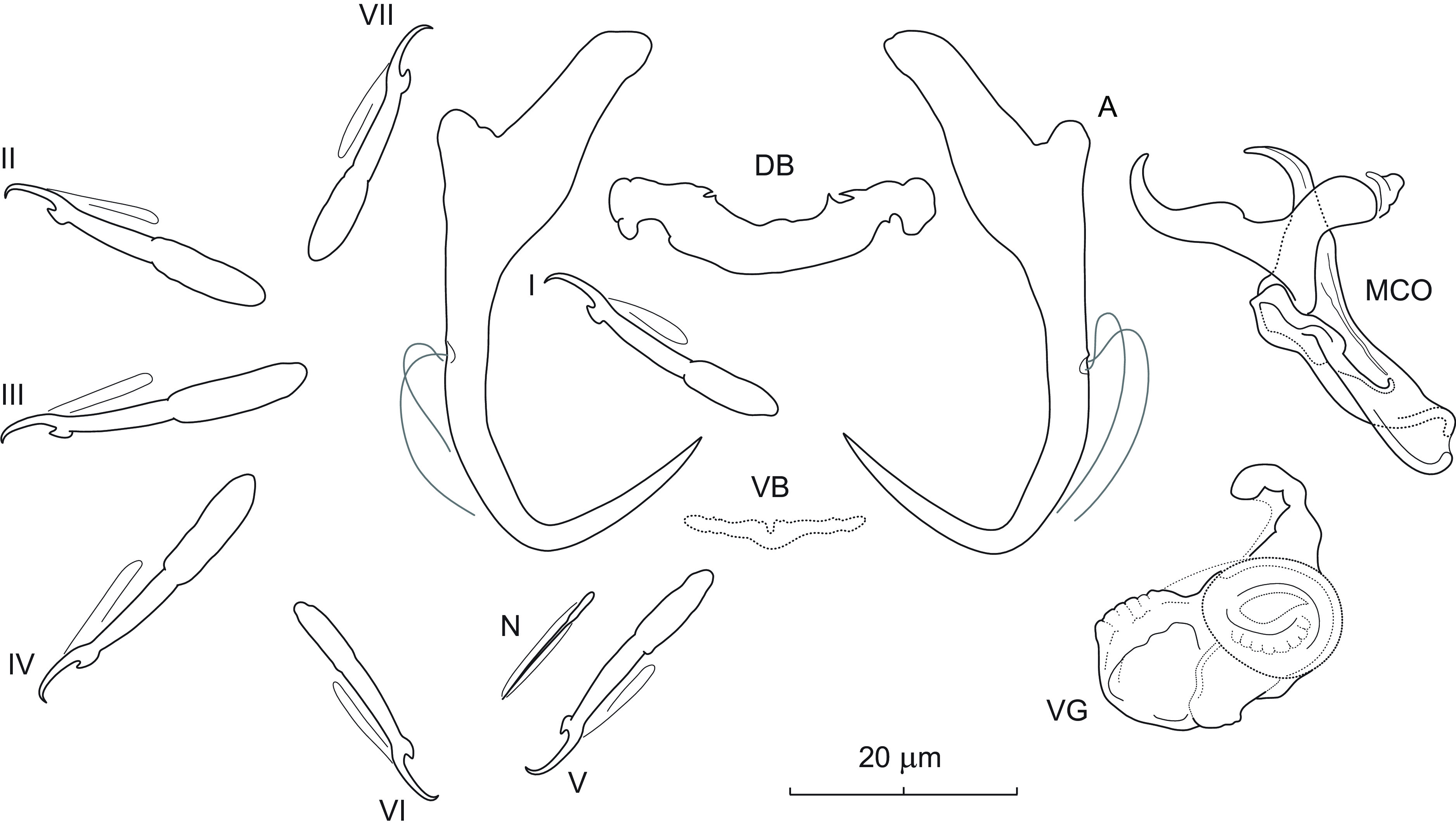
Sclerotized structures of *Dactylogyrus haneki* n. sp. ex *Hypentelium nigricans*. A – anchor; DB – dorsal bar; VB – ventral bar; N – needle; I–VII – hooks; VG – vagina; MCO – male copulatory organ.

Synonym: *Dactylogyrus* sp. 6 sensu Šimková et al. [82].

*Type host*: *Hypentelium nigricans* (Lesueur, 1817), Catostomidae (Catostominae).

*Type locality*: Arkansas, Huddleston Creek (Montgomery Co.).

*Other locality*: Arkansas, Walnut Creek (Garland Co.).

*Site on host*: Gill lamellae.

*Prevalence and intensity of infection*: 67% (2 fish infected/3 fish examined); 2–4 monogeneans per infected host.

*Type-specimens and specimens deposited*: Holotype (MNHN HEL1947); one paratype (MNHN HEL1948); two hologenophores (MNHN HEL1977-1978).

*Etymology*: This species is named after Dr. George Hanek in recognition of his contributions to the systematics and taxonomy of monogeneans parasitizing fishes in Canada.

*Comparative material studied*: *D. acicularis* Rogers, 1967 (USNM 061368; two paratypes) from *Moxostoma poecilurum*; *D. apos* Mueller, 1938 (USNM 071443; three cotypes) from *H. nigricans*.

*Description* (based on 2 specimens in GAP and 4 hologenophores): Anchors with elongate inner root (approx. 4 times the length of outer root) having slightly flattened termination, rounded outer root, medially slightly constricted bent shaft, and straight sharply recurved point extending past level of tip of inner root. Dorsal bar yoke-shaped, with subterminal bilateral notches on posterior border. Ventral bar vestigial, rod-shaped, with slight posteromedial expansion and irregular margins. One pair of needles located near hooks of pair V. Hooks with delicate point, upright acute thumb, shank comprised of 2 subunits (proximal expansion about 0.5 shank length); FH loop extending nearly to union of shaft subunits. MCO massive, composed of basally articulated copulatory tube and accessory piece. Copulatory tube with markedly elongate base and short arched shaft. Accessory piece broadly V-shaped, with short base bifurcated into two unequal rami; left ramus longer, claw-shaped, submedially with hump on inner surface; right ramus shorter, rounded, distally with lightly sclerotized joint-like extension*.* Vagina a massive plate-like structure with irregular margins, occasionally with centrally situated part resembling snail operculum.

*Measurements*: Body 433 (399–468; *n* = 2) long; greatest width 65 (61–69; *n* = 2). Haptor 88 (80–96; *n* = 2) long, 115 (103–127; *n* = 2) wide. Anchor: total length 46 (43–49; *n* = 5); inner root length 15 (13–18; *n* = 5); outer root length 4 (4–5; *n* = 5); point length 17 (16–18; *n* = 5). Dorsal bar 31 (29–34; *n* = 5) long. Ventral bar 15 (14–16; *n* = 5) long. Hooks (I–VII) 27 (21–34; *n* = 5) long: pair I 25 (24–28), pair II 27 (25–29), pair III 31 (28–34), pair IV 29 (27–33), pair V 26 (24–29), pair VI 24 (21–26), and pair VII 24 (23–27). MCO: total straight length 41 (38–46; *n* = 5); tube trace length 23 (21–24; *n* = 5). Vagina: greatest length 23 (15–29; *n* = 5); greatest width 21 (13–25; *n* = 5).

*Remarks*: *Dactylogyrus haneki* n. sp. was collected from the gills of the northern hog sucker (*H. nigricans*) in association with *D. chieni* n. sp. Morphologically, *D. haneki* n. sp. belongs to the same group as the latter species, and appears most similar to *D. acicularis* (from *Moxostoma poecilurum* Jordan, Alabama and Louisiana; [72]) and *D. apos* (from *H. nigricans*, New York; [57]) by having an accessory piece with a well-defined short base and two rami bifurcated almost perpendicularly to the copulatory tube with its shaft passing between them. *Dactylogyrus haneki* n. sp. is differentiated from both species by having the right ramus of the accessory piece with a rounded joint-like termination (the corresponding ramus in *D. acicularis* and *D. apos* having a pointed or recurved termination). *Dactylogyrus haneki* n. sp. differs further from *D. acicularis* by having an accessory piece with rami at an angle of 170° to each other (*vs* 90° to each other in *D. acicularis*) and from *D. apos* by having the left ramus of the accessory piece with a claw-shaped termination (the left ramus having a two-pointed termination in the latter species).

### *Dactylogyrus eos* Hanek, Molnár & Fernando, 1975 (Fig. 8)

Synonym: *Dactylogyrus* sp. 11 sensu Šimková et al. [82].

**Figure 8:**
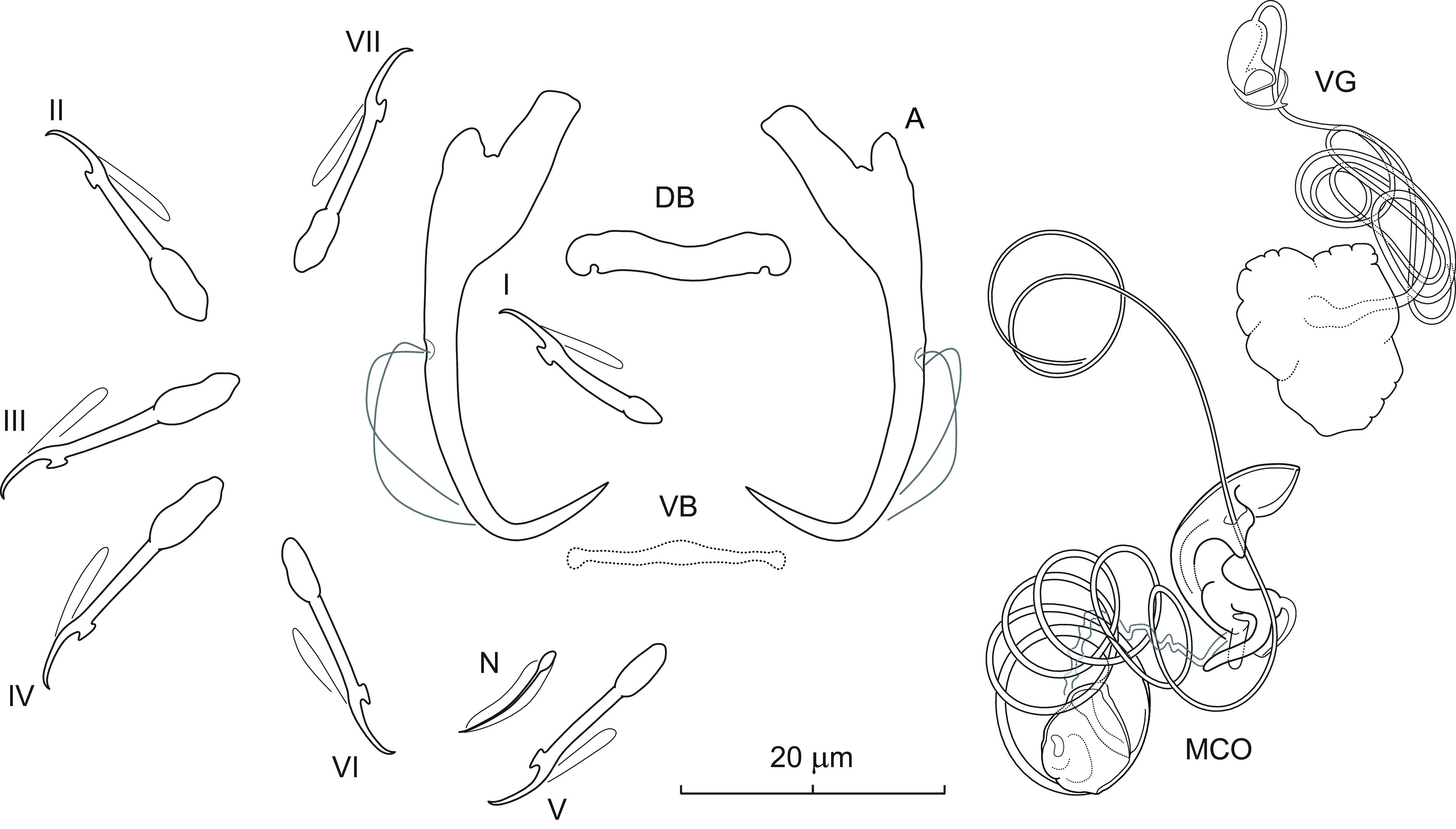
Sclerotized structures of *Dactylogyrus eos* Hanek, Molnár & Fernando, 1975 ex *Chrosomus neogaeus*. A – anchor; DB – dorsal bar; VB – ventral bar; N – needle; I–VII – hooks; VG – vagina; MCO – male copulatory organ.

*Type host*: *Chrosomus eos* Cope, 1861; Leuciscidae (Laviniinae).

*Type locality*: Ontario, Saugeen River (Durham).

*Previous records*: *Chrosomus eos* (syn. *Phoxinus eos*): Ontario [24, 32], New Brunswick [21].

*Current record*: *Chrosomus neogaeus* (Cope, 1867): Wisconsin, Mink River (Door Co.).

*Site on host*: Gill lamellae.

*Prevalence and intensity of infection*: 23% (3 fish infected/13 fish examined); 1–3 monogeneans per infected host.

*Specimens deposited*: Morphological voucher (MNHN HEL1950); one hologenophore (MNHN HEL1974).

*Comparative material examined*: *D. eos* (USNM 73154, No. 5) from *C. eos*.

*Redescription* (based on 3 specimens in GAP and 3 hologenophores): Anchors with moderately long inner root (approximately 2.5 times the length of outer root) having flattened termination, well-developed rounded inner root, elongate shaft forming gentle arc, and straight recurved point extending just past level of tip of inner root. Dorsal bar rod-shaped, with small subterminal notches on posterior border. Ventral bar vestigial, rod-shaped, with slight anteromedial expansion and enlarged ends. One pair of needles located near hooks of pair V. Hooks with delicate point, upright thumb, shank comprised of 2 subunits (proximal expansion less than 0.5 shank length); FH loop about 3/4 length of distal portion of shank. MCO composed of copulatory tube and accessory piece. Copulatory tube a coil of about six rings, with bulbous base. Accessory piece basally articulated to tube base; proximal part a zigzag rod lying within tube rings; distal part resembling an orchid labellum, medially bent at about a right angle, serving as a guide for protruding part of the copulatory tube during copulation*.* Vagina dextral; composed of a massive plate-like structure, long tortuous vaginal canal, and capsule-shaped distal opening.

*Measurements*: Body 384 (321–447; *n* = 3) long; greatest width 42 (42–43; *n* = 3). Haptor 50 (46–55; *n* = 3) long, 69 (67–70; *n* = 3) wide. Anchor: total length 34 (33–35; *n* = 3); inner root length 8 (8–9; *n* = 3); outer root length 3 (2–3; *n* = 3); point length 8 (7–8; *n* = 3). Dorsal bar 16 (16–17; *n* = 3) long. Ventral bar 17 (16–17; *n* = 3) long. Hooks (I–VII) 18 (14–22; *n* = 3) long: pair I 15 (14–16), pair II 18 (17–19), pair III 20 (20–21), pair IV 20 (18–22), pair V 18 (18–19), pair VI 18 (17–18), and pair VII 19 (18–20). MCO: total straight length 29 (27–31; *n* = 1); proximal ring diameter 12 (10–13; *n* = 3). Vagina: plate-like structure 15 (13–17; *n* = 3) in greatest diameter.

*Remarks*: *Dactylogyrus eos* is the only *Dactylogyrus* species hitherto reported from Nearctic cypriniforms that has a spirally-coiled copulatory tube. This species was described and later reported from *Chrosomus eos* (syn. *Phoxinus eos*) in Ontario [24, 32] and New Brunswick [21]. The monogeneans collected from *C. neogaeus* during the present study generally correspond to the original description of *D. eos*, but there are some uncertainties mainly concerning the hard reproductive structures (see below).

Although Hanek *et al.* [32] stated that two paratypes of *D. eos* were deposited in the USNM, our examination showed that one of them (USNM 73154, No. 6) is represented by the second *Dactylogyrus* species reported on *C. eos* as well as *C. neogaeus*, namely *D. chrosomi* Hanek, Molnár & Fernando, 1975 [23, 24, 32]. In addition, unfortunately, the only available paratype of *D. eos* (USNM 73154, No. 5) was contracted and highly transparent. In the original description, Hanek *et al.* [32] indicated that *D. eos* possesses an MCO with four coils and that the vagina is muscular, which does not correspond to our specimens. The presence of a long tortuous vaginal canal observed in our specimens from *D. neogaeus* could not be confirmed with certainty in the paratype of *D. eos*; however, a suggestion of this structure and the presence of a massive plate-like vaginal part are apparent in the paratype. As for the number of rings of the copulatory tube in our specimens from *D. neogaeus*, it should be mentioned that only a single specimen was suitable for accurately detecting the course of individual rings. In this specimen, however, a considerable distal portion of the tube appeared to be unwound from the accessory piece, as seen in Figure 8. The spiral nature of the copulatory tube and its basal articulation with the proximal part of the accessory piece lying within the tube coil, along with the copulatory tube partly protruding from the genital pore (or accessory piece) was also observed by Kritsky *et al.* [40] in specimens of *Dawestrema* spp. (Dactylogyridae). These authors suggested that the accessory piece in species of *Dawestrema* may function similarly to the spring mechanism of a self-retracting tape measure. As in species of *Dawestrema*, the diameter of the tube rings in our specimens from *C. neogaeus* does not appear to be affected by the extrusion of the terminal part of the tube. In accordance with Kritsky *et al.* [40], we assume that the protrusion of the copulatory tube during copulation is not the result of a tightening of the tube coil, but rather an unwinding of the tube shaft with the assistance of the proximal part of the accessory piece. However, confirmation of this mechanism will require further investigation, ideally based on the observation of living worms. In view of the above, the course of the copulatory tube is probably somewhat variable and its strict number of coils does not appear to be a consistent character for the identification of this species. Differences observed in the anchors between the paratype of *D. eos* and our specimens (*i.e.*, a slightly longer inner root compared with the outer root in the paratype) were minimal and fall within expected intraspecific variation among *Dactylogyrus* species, especially when specimens originate from two different host species and localities. Finally, although Hanek *et al.* [32] did not mention the presence of the ventral bar and although this structure was not detectable even in the paratype due to its poor condition, in the present specimens from *C. neogaeus* the ventral bar is visible, albeit poorly, and it is therefore possible that Hanek *et al.* [32] overlooked its presence in their four specimens.

Thus, on the basis of the above, we tentatively consider the six *Dactylogyrus* specimens from *C. neogaeus* to represent *D. eos*. However, confirmation of this identification will depend on the collection and examination of new parasite material from *Chrosomus eos* from or near the type locality in Ontario.

### *Dactylogyrus fimbratus* n. sp. (Fig. 9)

urn:lsid:zoobank.org:act:65EF6712-AB73-40FD-B3B3-9847368AF8F5

**Figure 9:**
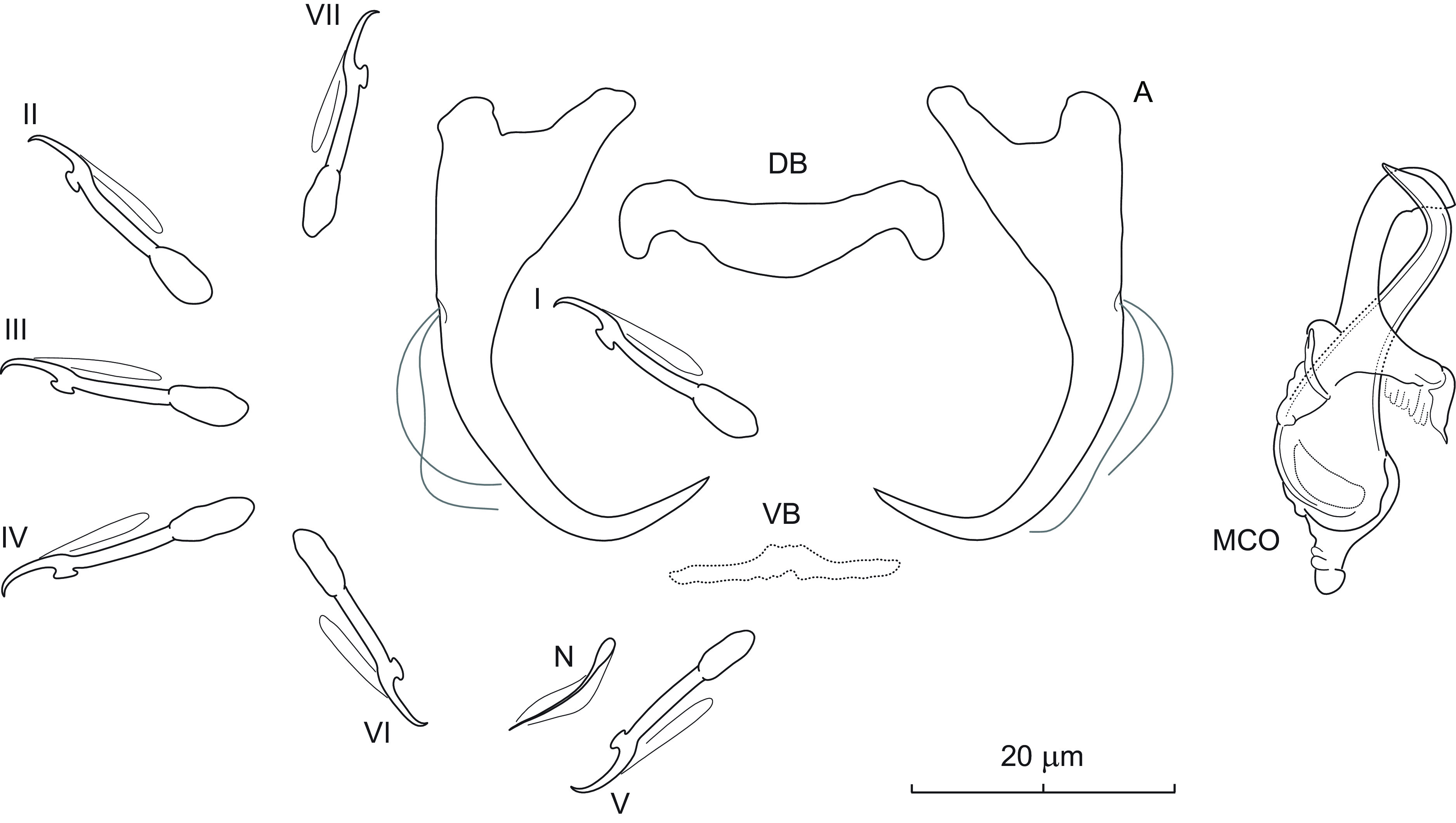
Sclerotized structures of *Dactylogyrus fimbratus* n. sp. ex *Rhinichthys cataractae*. A – anchor; DB – dorsal bar; VB – ventral bar; N – needle; I–VII – hooks; MCO – male copulatory organ.

Synonym: *Dactylogyrus* sp. 10 sensu Šimková et al. [82].

*Type host*: *Rhinichthys cataractae* (Valenciennes, 1842), Leuciscidae (Pogonichthyinae).

*Type locality*: New York, Leatherstocking Creek (Otsego Co.).

*Site on host*: Gill lamellae.

*Prevalence and intensity of infection*: 20% (1 fish infected/5 fish examined); 3 monogeneans per infected host.

*Type-specimens and specimens deposited*: Holotype (MNHN HEL1946); one hologenophore (MNHN HEL1975).

*Etymology*: The specific name (an adjective) is from Latin (*fimbratus* = fringed) and refers to the accessory piece of the MCO.

*Description* (based on two specimens in GAP and one hologenophore): Anchors with short inner root (approx. 1.5 times the length of outer root) having slightly flattened termination, broad outer root, bent shaft, and point extending past level of tip of inner root. Dorsal bar yoke-shaped. Ventral bar vestigial, rod-shaped, with anteromedial expansion. One pair of needles located near hooks of pair V. Hooks with delicate point, upright acute thumb, shank comprised of 2 subunits (proximal expansion about 0.75 shank length); FH loop extending nearly to union of shaft subunits. MCO composed of basally articulated copulatory tube and accessory piece. Copulatory tube with relatively large base extended posteriorly into the shape of a dwarf’s hat; shaft distally slightly recurved, terminally truncated. Accessory piece inverted T-shaped, with swollen recurved distal end; proximal part partly fringed, resembling a wing. Vagina not observed.

*Measurements*: Body 380 (*n* = 1) long; greatest width 75 (*n* = 1). Haptor 58 (*n* = 1) long, 73 (*n* = 1) wide. Anchor: total length 35 (34–35; *n* = 2); inner root length 7 (7–8; *n* = 2); outer root length 3 (*n* = 2); point length 9 (8–10; *n* = 2). Dorsal bar 24 (*n* = 2) long. Ventral bar 18 (17–18; *n* = 2) long. Hooks (I–VII) 18 (17–20; *n* = 2) long: pair I 18, pair II 18, pair III 19 (18–19), pair IV 20, pair V 18, pair VI 18 (17–18), and pair VII 17 (16–17) long. MCO: total straight length 32 (32–33; *n* = 3); tube trace length 34 (*n* = 3).

*Remarks*: On the basis of similarities of the respective haptoral elements and MCOs, *Dactylogyrus fimbratus* n. sp. resembles *Dactylogyrus cheloideus* Rogers, 1967 described from *Rhinichthys atratulus* (Hermann) in Alabama [72] and later reported on the same host in Ontario by Hanek and Furtado [31] as *D. atratuli* (=synonym for *D. cheloideus*; [39]). Both species possess roots of similar size (*i.e.*, the inner root is only slightly longer than the outer root), a relatively robust yoke-shaped dorsal bar, and a vestigial ventral bar with anteromedial expansion. In addition, the distal ends of the copulatory tube and accessory piece curve inward to give them a pincer-like appearance in both species. *Dactylogyrus fimbratus* n. sp. clearly differs from *D. cheloideus* by having (i) a copulatory tube with a bulbous base extended posteriorly into the shape of a dwarf’s hat (the base reduced in size and truncated in *D. cheloideus*) and a slightly longer and slender shaft, and (ii) an accessory piece with a swollen distal end (*vs* a tapered distal end in *D. cheloideus*) and a partly fringed proximal part (without fringes in *D. cheloideus*). Although only three specimens of this species were collected from longnose dace, the unique features of the MCO make them sufficiently distinct from all known congeners to be considered as representing a new species of *Dactylogyrus*.

### *Dactylogyrus parvicirrus* Seamster, 1948 (Fig. 10)

Synonym: *Dactylogyrus cf. parvicirrus* sensu Šimková et al. [82].

**Figure 10:**
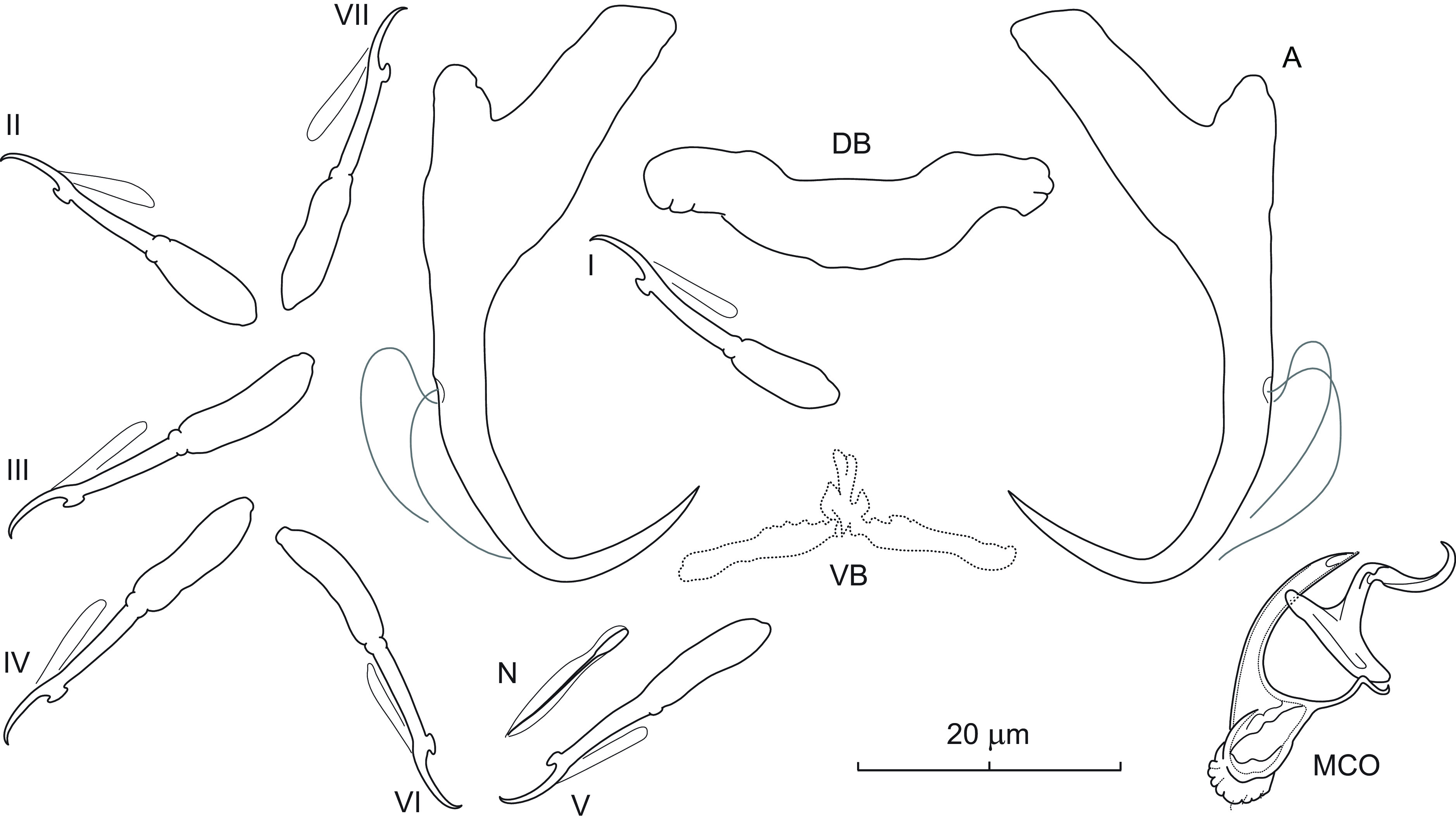
Sclerotized structures of *Dactylogyrus parvicirrus* Seamster, 1948 ex *Notemigonus crysoleucas*. A – anchor; DB – dorsal bar; VB – ventral bar; N – needle; I–VII – hooks; MCO – male copulatory organ.

*Type host*: *Notemigonus crysoleucas* (Mitchill, 1814), Leuciscidae (Leuciscinae).

*Type locality*: Oklahoma; Culwell’s Pond (Muskogee Co.).

*Previous records*: *Notemigonus crysoleucas*: Oklahoma [78], Virginia [33], Texas [60], Alabama [72], Ontario [22, 24], New Brunswick [21].

*Current record*: *Notemigonus crysoleucas*: New York, Rom Hill Beaver Pond (Otsego Co.).

*Site on host*: Gill lamellae.

*Prevalence and intensity of infection*: 100% (5 fish infected/5 fish examined); 1–8 monogeneans per infected host.

*Specimens deposited*: Three morphological vouchers (MNHN HEL1951-1952); two hologenophores (MNHN HEL1987-1988).

*Redescription* (based on 7 specimens in GAP and 3 hologenophores): Anchors with elongate inner root (approx. 3 times the length of outer root) having flattened termination, well-developed outer root, medially slightly constricted bent shaft, and straight recurved point extending to level of tip of inner root. Dorsal bar yoke-shaped, massive. Ventral bar with irregular margins and frayed anteromedial projection. One pair of needles located near hooks of pair V. Hooks with delicate point, small upright thumb, shank comprised of 2 subunits (proximal expansion about 0.5 shank length); FH loop about 3/4 length of distal portion of shank. MCO small in relation to size of haptoral structures, comprises copulatory tube articulated to accessory piece by articulation process. Copulatory tube with funnel-shaped base surrounded by flange and filamentous articulation process arising from inner side of the base; shaft arched, with tapered distal end. Accessory piece a plate with anteromedial S-shaped arm, medially looping around distal termination of the copulatory tube. Vagina not observed.

*Measurements*: Body 293 (250–320; *n* = 3) long; greatest width 66 (53–76; *n* = 3). Haptor 55 (43–64; *n* = 3) long, 67 (52–82; *n* = 3) wide. Anchor: total length 41 (37–44; *n* = 5); inner root length 13 (11–15; *n* = 5); outer root length 5 (4–6; *n* = 5); point length 12 (10–12; *n* = 5). Dorsal bar 29 (24–31; *n* = 5) long. Ventral bar 24 (21–26; *n* = 5) long. Hooks (I–VII) 24 (19–28; *n* = 5) long: pair I 22 (19–23), pair II 24 (21–28), pair III 26 (23–28), pair IV 25 (21–28), pair V 24 (22–25), pair VI 24 (22–26), pair VII 24 (21–26). MCO: total straight length 22 (20–26; *n* = 5); tube trace length 20 (19–21; *n* = 5).

*Remarks*: *Dactylogyrus parvicirrus* covers a comparatively large geographical range, having been recorded from a total of five states (including the present study) and two provinces in the United States and Canada, respectively (see Taxonomic summary). This species is easily distinguished from its North American congeners by its unique MCO, in which the accessory piece has the shape of the letter T with its stem twisted around the distal half of the copulatory tube. However, the original drawings by Seamster [78] are highly diagrammatic (probably drawn free hand instead of from a camera lucida), and, unfortunately, the type specimens probably do not exist. Although Seamster [78] indicates that cotypes were deposited in the University of Notre Dame type collection, Indiana, there is no record of them in the collection (Barbara Hellenthal, personal communication). Present respective measurements of the haptoral structures and the MCO generally correspond to those in the original description, but there are discrepancies concerning the presence/absence of the ventral bar between the current specimens and the original account of *D. parvicirrus*. In his description based on twelve specimens, Seamster [78] stated that the ventral bar was not observed; also, Hargis [33] did not depict the ventral bar in his drawings from specimens collected in Virginia. However, in all of the seven specimens available to us, the ventral bar was clearly visible (see Fig. 10). This is in accordance with the phase contrast microscopy observations of Mizelle and Price [54], who depicted the haptoral parts of *D. parvicirrus* with a ventral bar morphologically corresponding to that in our specimens. The ventral bar, if present, is differentially developed and not always clearly visible in fixed specimens of *Dactylogyrus* spp. (*e.g.*, [52]). Thus, it is possible that Seamster [78] and Hargis [33] missed the presence of the ventral bar because they did not use phase contrast optics. In addition, both authors mounted their parasites in a glycerin-gelatin medium, which makes the specimens more transparent. Another possibility is that the variation in the morphology/development of the ventral bar among *D. parvicirrus* populations from various localities can be attributed to intraspecific variation.

The general configuration of the MCO in the current specimens corresponds well with drawings made by both Seamster [78] and Hargis [33]. The only feature by which our specimens differ from the previous illustrations is the presence of a thin filament arising from the base of the copulatory tube and articulated to the plate-like part of the accessory piece. This difference may have arisen due to the different techniques used to mount the worms by the aforementioned authors and to the fact that this structure is clearly visible only under phase contrast optics. In view of the above, we tentatively consider the specimens from *N. crysoleucas* collected in New York to represent *D. parvicirrus*. Confirmation of this species identification will depend on the collection and examination of supplementary monogenean material (morphological and DNA samples) from or near the type locality in Oklahoma.

### Phylogenetic relationships and morphology of Nearctic *Dactylogyrus* species

**Figure 11:**
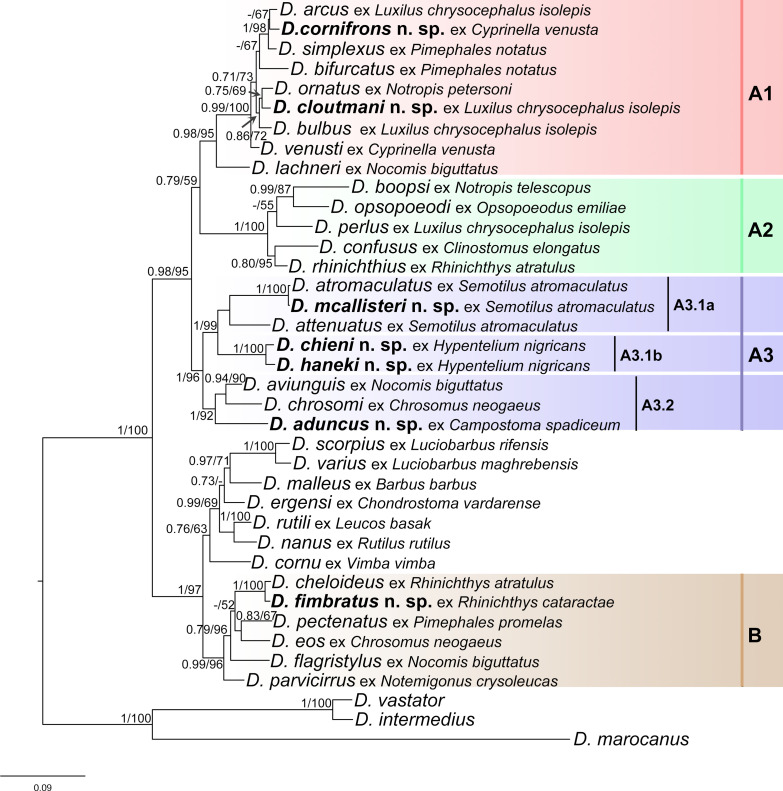
Phylogenetic tree of 38 *Dactylogyrus* species from Nearctic and Palaearctic cypriniforms resulting from BI analysis. The tree is based on concatenated partial sequences of the 28S rDNA, 18S rDNA, and ITS1 regions. Numbers along branches indicate posterior probabilities and bootstrap values resulting from BI and ML analyses, respectively. Only values >0.70 for BI and >50% for ML are shown. The new species of *Dactylogyrus* are highlighted in bold.

A total of 28 species of *Dactylogyrus* (*i.e.*, seven new and 21 previously described) parasitizing Nearctic cypriniforms were used for molecular phylogenetic reconstruction in order to investigate the link between their phylogenetic relationships and the morphological characters of the haptor and MCO. Bayesian inference and maximum likelihood analyses, based on concatenated data sets of partial 28S rDNA, partial 18S rDNA and ITS1, generated phylogenetic trees with congruent topologies; therefore, only the BI tree is presented (Fig. 11). *Dactylogyrus* species parasitizing Nearctic cypriniforms were positioned in two main well-supported clades (A and B). Clade A comprises 22 strictly Nearctic *Dactylogyrus* species and was composed of three subclades (A1–A3). Clade B includes six Nearctic *Dactylogyrus* species in sister position to a clade with *Dactylogyrus* spp. from European leuciscids and North-West African cyprinids. Strictly Nearctic species of *Dactylogyrus* (clade A) share the same basic MCO morphology (=nearctic type) – however, with minor modifications typical for each monophyletic group (see below). In contrast, Nearctic *Dactylogyrus* species forming clade B possess an MCO of diverse morphology.

**Figure 12:**
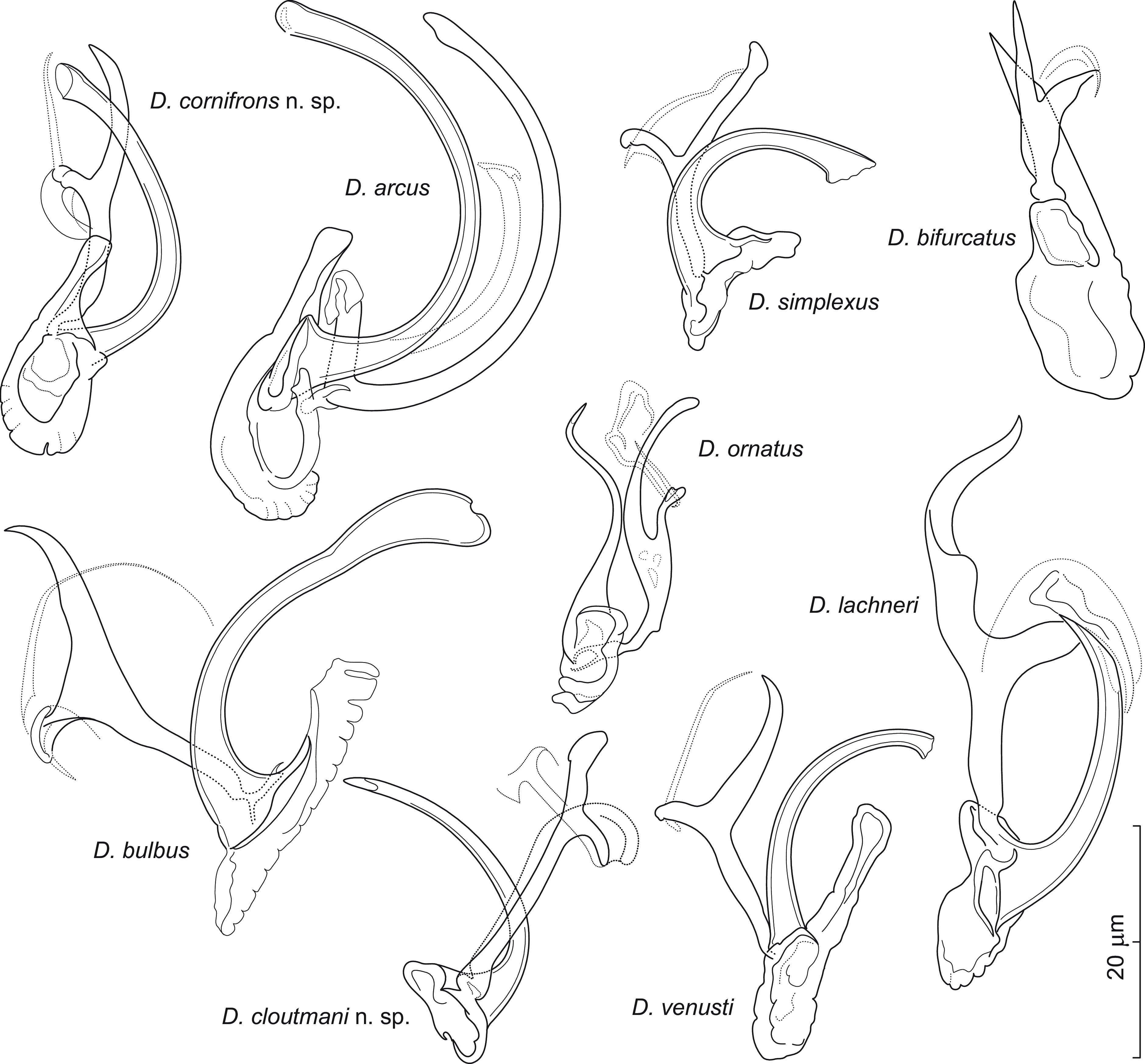
Drawings of the MCOs taken from hologenophores of *Dactylogyrus* spp. included in subclade A1 of the phylogenetic tree (Fig. 11). *Dactylogyrus arcus* ex *Luxilus chrysocephalus isolepis* (Arkansas), *D. bifurcatus* ex *Pimephales notatus* (Arkansas), *D. bulbus* ex *Luxilus c. isolepis* (Arkansas), *D. cloutmani* n. sp. ex *Luxilus c. isolepis* (Arkansas), *D. cornifrons* n. sp. ex *Cyprinella venusta* (Mississippi), *D. lachneri* ex *Nocomis biguttatus* (Wisconsin), *D. ornatus* ex *Notropis petersoni* (Mississippi), *D. simplexus* ex *P. notatus* (Arkansas), *D. venusti* ex *C. venusta* (Mississippi).

The first subclade (A1) was well-supported by both BI and ML analyses and includes nine species of *Dactylogyrus* parasitizing fish species of Pogonichthyinae. *Dactylogyrus lachneri* from *N. biguttatus* is in basal position to the remaining species of this subclade, followed by *D. venusti* from *C. venusta*, and a group comprising seven species of *Dactylogyrus* from hosts representing four different genera (*Cyprinella*, *Luxilus*, *Notropis*, and *Pimephales*, see Fig. 11). Although the phylogenetic relationships among the seven species are mostly either weakly resolved or unresolved, all of them share similar morphology with respect to their haptoral structures (see *D. cornifrons* n. sp. for example, Fig. 3). These are characterized by (i) anchors with moderately developed roots, an elongate shaft having a median constriction, and a well-differentiated point forming an acute angle with the shaft; (ii) a dorsal bar that is straight or slightly bent posteriorly, with slightly enlarged ends; and (iii) an inverted V-shaped ventral bar with an anteromedial knob. Unlike *D. venusti*, which possesses the same haptoral configuration as the seven species, *D. lachneri* clearly differs from the remaining species in subclade A1 by a saddle-shaped ventral bar lacking an anteromedial knob. All *Dactylogyrus* species representing subclade A1 share a similar morphology of the MCO (Fig. 12), *i.e.*, a relatively simple copulatory tube supported by a bifurcated accessory piece, but there are group-forming differences in features concerning the copulatory tube. As for the base of the copulatory tube, in four species (*D. arcus*, *D. cornifrons* n. sp., *D. lachneri*, and *D. venusti*), the flange surrounding the base opening protrudes into an anteriorly directed heel, while in the other species the heel is lacking. The shape of the copulatory tube of the species belonging to subclade A1 varies from straight (*D. bifurcatus*) through arched (*D. arcus*, *D. bulbus*, *D. cloutmani* n. sp., *D. cornifrons* n. sp., *D. lachneri*, *D. simplexus*, and *D. venusti*) to sinuous (*D. ornatus*). The last differences concern the form of the distal termination of the copulatory tube, which may be slightly enlarged or flared (*D. arcus*, *D. bulbus*, *D. cornifrons* n. sp., *D. simplexus*, *D. venusti*), tapering (*D. cloutmani* n. sp., *D. ornatus*) or diagonally truncated (*D. bifurcatus*, *D. lachneri*).

**Figure 13:**
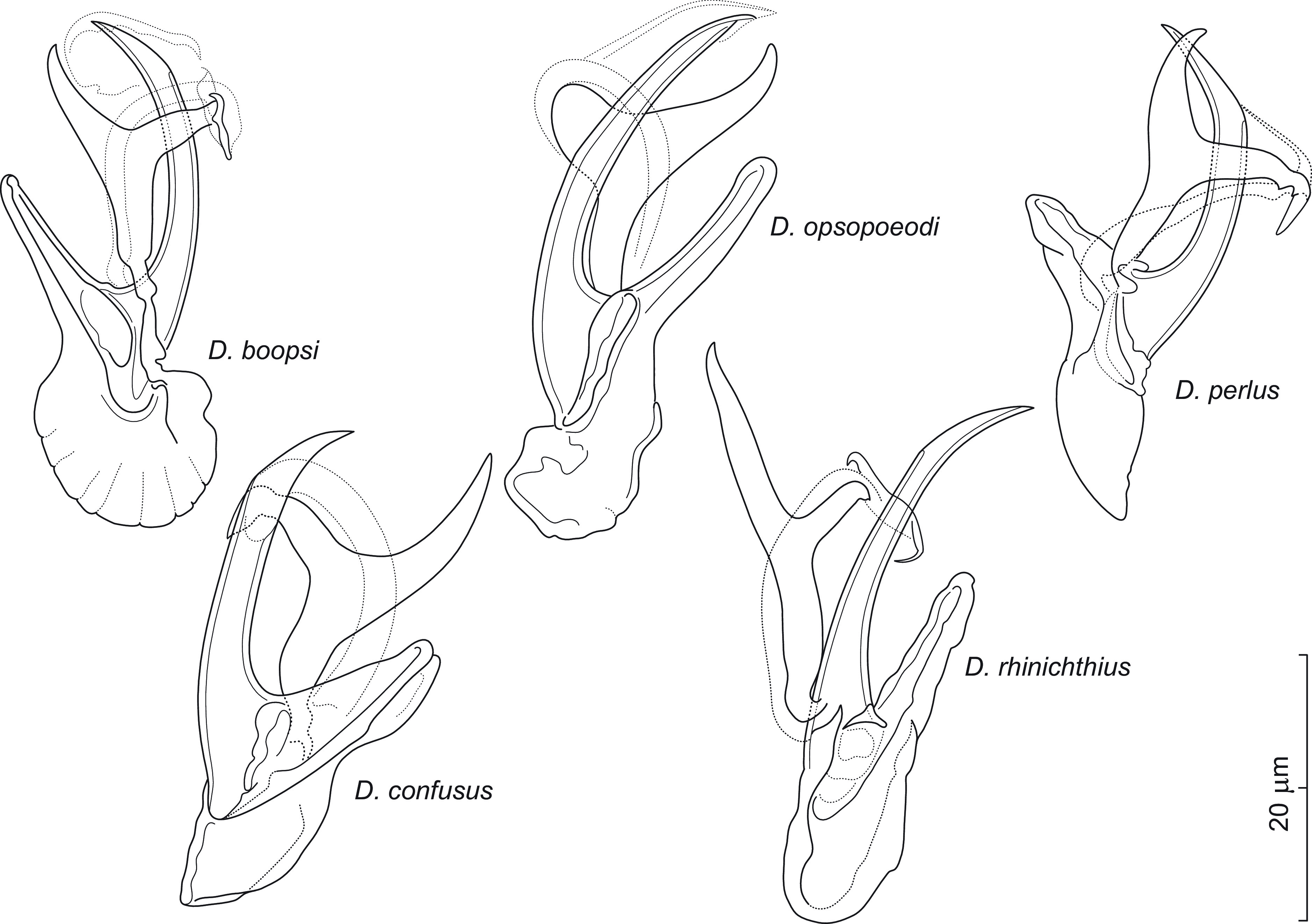
Drawings of the MCOs taken from hologenophores of *Dactylogyrus* spp. included in subclade A2 of the phylogenetic tree (Fig. 11). *Dactylogyrus boopsi* ex *Notropis telescopus* (Arkansas), *D. confusus* ex *Clinostomus elongatus* (Wisconsin), *D. opsopoeodi* ex *Opsopoeodus emiliae* (Mississippi), *D. perlus* ex *Luxilus chrysocephalus isolepis* (Arkansas), *D. rhinichthius* ex *Rhinichthys atratulus* (Wisconsin).

The second subclade (A2) is strongly supported by posterior probabilities (PP) and bootstrap (BP) values from BI and ML analyses, respectively, and comprises five *Dactylogyrus* species parasitizing five species of Pogonichthyinae, each representing a different genus (*i.e.*, *Clinostomus*, *Luxilus*, *Notropis*, *Opsopoeodus*, and *Rhinichthys*). All of these *Dactylogyrus* species share similar haptoral structures (see *D. perlus* for example, Fig. 4), *i.e.*, anchors with a point having a recurved tip and forming an approximate right angle with a relatively short shaft, a broadly V-shaped dorsal bar with a medial membrane, and a straight vestigial ventral bar with a slight medial expansion. However, in *D. rhinichthius*, the ventral bar is absent and the dorsal bar is straight rather than V-shaped. *Dactylogyrus* spp. included in subclade A2 share similar morphology of the MCO (Fig. 13) with *D. lachneri* (with a small modification of the left ramus) and some species in the neighboring subclade A3 (*i.e.*, *D. aviunguis* and *D. chrosomi*). The MCO is characterized by an accessory piece medially bifurcated into two unequal rami and a copulatory tube having: (i) a relatively robust base anteriorly enlarged into a heel, (ii) an almost straight to arched shaft, and (iii) a diagonally truncated distal end.

**Figure 14:**
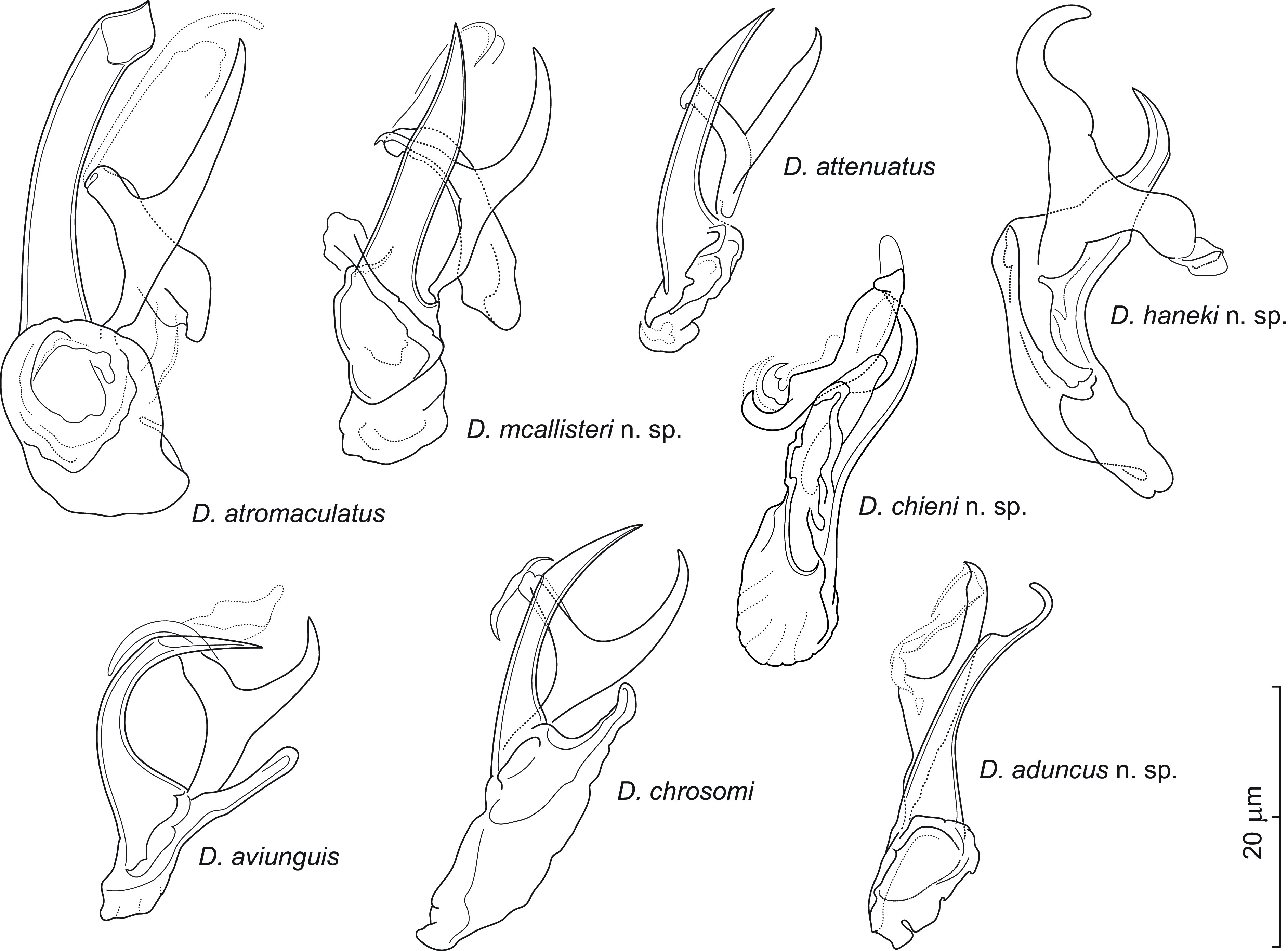
Drawings of the MCOs taken from hologenophores of *Dactylogyrus* spp. included in subclade A3 of the phylogenetic tree (Fig. 11). *Dactylogyrus aduncus* n. sp. ex *Campostoma spadiceum* (Arkansas), *D. atromaculatus* ex *Semotilus atromaculatus* (Wisconsin), *D. attenuatus* ex *S. atromaculatus* (Wisconsin), *D. aviunguis* from *Nocomis biguttatus* (Wisconsin), *D. chieni* n. sp. ex *Hypentelium nigricans* (Arkansas), *D. chrosomi* ex *Chrosomus neogaeus* (Wisconsin), *D. haneki* n. sp. ex *H. nigricans* (Arkansas), *D. mcallisteri* n. sp. ex *S. atromaculatus* (Arkansas).

The third well-supported subclade (A3) includes *Dactylogyrus* species of Leuciscidae and Catostomidae and is subdivided into two monophyletic groups A3.1 and A3.2. Group A3.1 comprises three *Dactylogyrus* spp. (*D. atromaculatus*, *D. mcallisteri* n. sp., and *D. attenuatus*) from *S. atromaculatus* (Leuciscidae, Plagopterinae) (=A3.1a) and two *Dactylogyrus* spp. (*D. chieni* n. sp. and *D. haneki* n. sp.) from *Hypentelium nigricans* (Catostomidae) (=A3.1b). These two monophyletic subgroups are morphologically well separated by both haptoral and reproductive hard structures (Fig. 14). Species from *S. atromaculatus* (A3.1a) are characterized by anchors with an evenly curved shaft and point, a straight dorsal bar with ends bevelled inwards, and a vestigial ventral bar with an anteromedial expansion (see Fig. 5); the MCO is characterized by (i) a copulatory tube with a base lacking an anteriorly directed heel and a short, slightly-arched shaft with a diagonally truncated distal end, and by (ii) a medially bifurcated accessory piece with the right ramus bearing a plate passing diagonally across the base of the accessory piece (less developed in *D. attenuatus*). The sister relationship of *D. atromaculatus* and *D. mcallisteri* n. sp. is supported by both haptoral and MCO similarities (see Remarks on *D. mcallisteri* n. sp.). Species from *H. nigricans* (A3.1b), *D. chieni* n. sp. and *D. haneki* n. sp., differ from the previous subgroup (A3.1a) by the shape of the anchors, which are of the same type as in most species forming subclade A1, by the morphology of the MCO, and by the presence of a sclerotized vagina. The huge elongate base, short arched shaft, and a broadly V-shaped accessory piece represent MCO characters by which *Dactylogyrus* spp. from *H. nigricans* clearly differ from those parasitizing Nearctic leuciscids. The well-supported monophyletic group A3.2 comprises *D. aviunguis* from *N. biguttatus* (Pogonichthyinae) and *D. chrosomi* from *C. neogaeus* (Laviniinae) (PP = 0.94; BP = 90), and *D. aduncus* n. sp. from *C. spadiceum* (Pogonichthyinae). Although *D. aviunguis* and *D. chrosomi* parasitize hosts of different subfamilies, these species share similar MCOs with *Dactylogyrus* spp. included in subclade A2.

**Figure 15:**
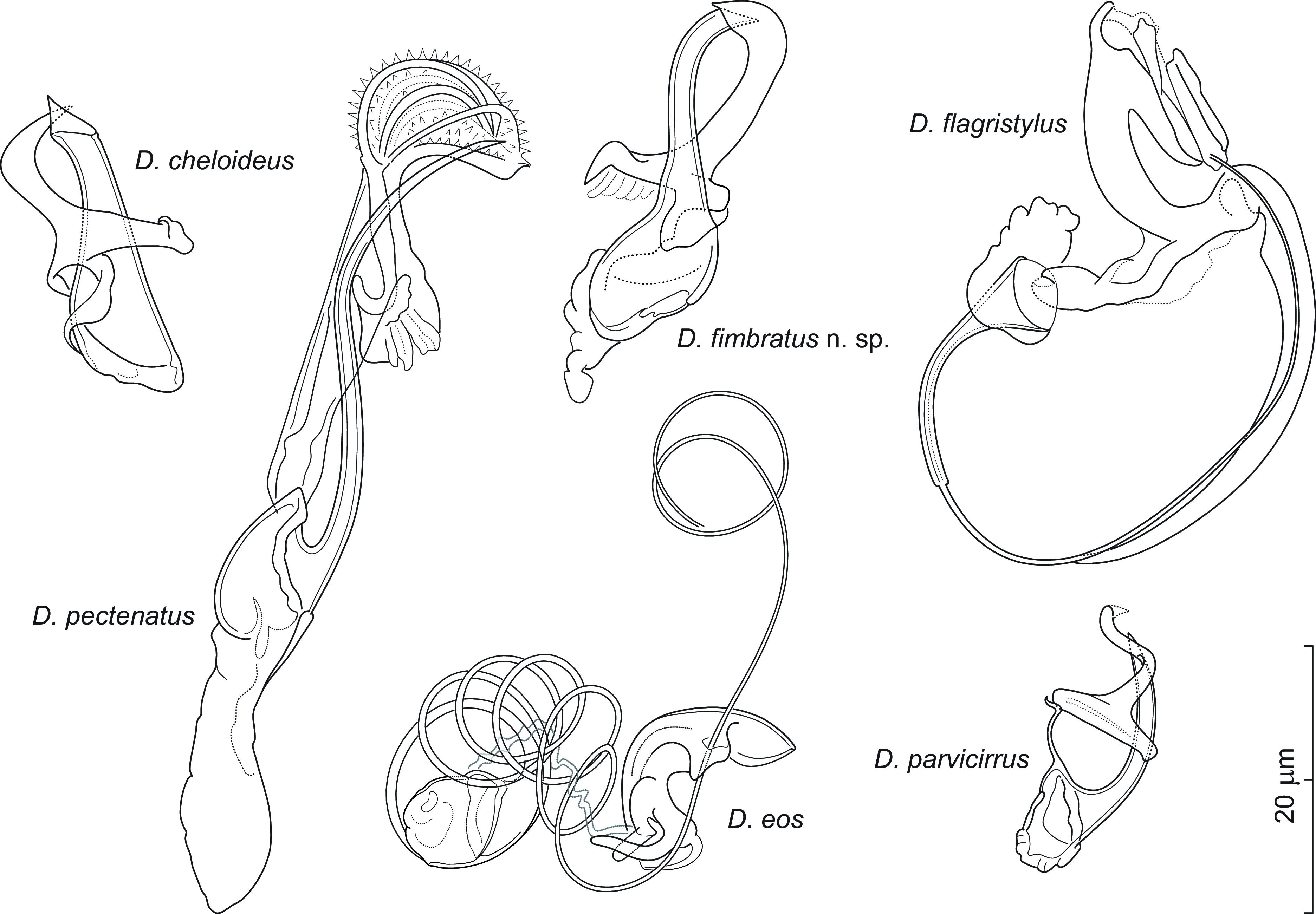
Drawings of the MCOs taken from hologenophores of *Dactylogyrus* spp. included in clade B of the phylogenetic tree (Fig. 11). *Dactylogyrus cheloideus* ex *Rhinichthys atratulus* (Wisconsin), *D. eos* ex *Chrosomus neogaeus* (Wisconsin), *D. fimbratus* n. sp. ex *Rhinichthys cataractae* (New York), *D. flagristylus* ex *Nocomis biguttatus* (Wisconsin), *D. parvicirrus* ex *Notemigonus crysoleucas* (New York), *D. pectenatus* ex *Pimephales promelas* (Wisconsin).

In the second main clade (B), species of *Dactylogyrus* parasitizing Nearctic leuciscids form a monophyletic group that is sister to *Dactylogyrus* spp. from European leuciscids and North-West African cyprinids. The phylogenetic analyses strongly support the phylogenetic proximity of two morphologically close species, namely *D. cheloideus* and *D. fimbratus* n. sp., both parasitizing species of *Rhinichthys*. However, their phylogenetic position to other *Dactylogyrus* spp. in clade B is weakly resolved only by ML analysis. *Dactylogyrus parvicirrus* from *N. crysoleucas*, the only host species representing Leuciscinae in the Nearctic region, appears to be sister to the remaining species in clade B that parasitize mostly Pogonichthyinae (with the exception of *D. eos* from *C. neogaeus* (Laviniinae)). The phylogenetic relationships among *D. flagristylus*, *D. eos*, and *D. pectenatus* were moderately or weakly supported by PP and BP in our analyses, which is consistent with the dissimilarity in the morphology of the hard structures (both haptoral and reproductive) of these species (Fig. 15).

## Discussion

Prior to the present study, 132 nominal species of *Dactylogyrus* had been recorded from 113 species of native cypriniform fishes in North America [19, 20, 41]. The seven new species described herein raise the number of formally described *Dactylogyrus* species to 139 and the number of known host species for *Dactylogyrus* spp. to 114. Given that more than half of North American species of *Dactylogyrus* are strict specialists parasitizing a single host species (61%; [41]), this number is undoubtedly an underestimate of the total diversity of these parasites, as more than 390 species of cypriniform fishes are currently known on this continent. Unfortunately, however, the descriptions of many of the previously described *Dactylogyrus* species from cypriniforms in North America are based on schematic illustrations and their taxonomic quality tends to be low. This makes species identification difficult and thereby increases the risk of duplicate descriptions, which indicates that some *Dactylogyrus* species may end up being synonyms.

The haptoral as well as reproductive hard structures in *Dactylogyrus* species express different morphological types, on the basis of which species of the genus may be grouped. These groups are often defined on the basis of the morphological type of one of these structures (*e.g.*, by anchor shape) and cannot be separated using all structures at once [3, 5, 66, 71, 80]. Given that the structural morphology and configuration of the attachment organ (*i.e.*, the haptor) are supposed to play an important role in monogenean specialization and adaptation to host species [42, 75, 79, 80], these morphological groups may be viewed as phylogenetic units connected with the host specificity of these parasites. However, the huge diversity in both haptoral and reproductive structures results in the categorization of many morphological groups in *Dactylogyrus* species (see [66]) and it is difficult to determine which structural characters indicate the degree of relatedness between different species of the genus. Moreover, as phenotypes are influenced by environmental factors as well as by genes, some morphological characters in *Dactylogyrus* species may undergo convergent evolution in divergent lineages [5, 66, 80]. Compared to the Palaearctic region, where many morphological groups of *Dactylogyrus* have been recognized on the basis of the morphological type of one particular structure [5, 66, 71], very little is known about morphological grouping within Nearctic species of *Dactylogyrus*. Chien [10–12] divided fifteen species of *Dactylogyrus* from *Nocomis* spp. into four groups: (i) the reciprocus group (*D. effusus*, *D. lachneri*, and *D. reciprocus*); (ii) the bellicus group (*D. ancylostylus*, *D. asper*, *D. aviunguis*, *D. latirictus*, *D. leptostylus*, *D. megastylus*, *D. millieae*, and *D. mississippiensis*); (iii) the limulus group (*D. flagristylus*, *D. limulus*, and *D. malleolus*), and (iv) the mollis group (*D. mollis*). He defined the above groups on the basis of a combination of morphological features, mainly those concerning the haptoral structures and MCO.

In the present study, 28 *Dactylogyrus* species parasitizing mostly species of Nearctic Leuciscidae and forming two phylogenetic clades with different origins [82] are divided into two main morphological groups on the basis of the MCO. Strictly Nearctic species of *Dactylogyrus* (clade A) share the same basic MCO morphology (=the nearctic morphological type) – however, with minor modifications typical of each phylogenetic subgroup. In contrast, Nearctic species forming a sister clade (clade B) to Palaearctic species of *Dactylogyrus* possess an MCO of diverse morphology. The “nearctic” type of MCO basically corresponds to that of *D. intermedius* and *D. vastator* (*i.e.*, “anchoratus” type in [66]), two species forming a basal group to all Nearctic *Dactylogyrus* spp. according to currently available DNA sequences, and parasitizing cyprinids with a Euro-Asian distribution ([82], present study). The largely accepted view is that fishes of Cyprinoidei originated in the Oriental region [2, 6]. Fossil records together with results of phylogenetic analyses showing the Far East Asian Pseudaspininae to be a sister group to all remaining leuciscids suggest a long history of cyprinoids in eastern Asia, and support the hypothesis that ancestral Asian leuciscids colonized North America *via* the Bering Land Bridge during the mid-Oligocene [8, 77]. The Asian origin of the clade comprising *Dactylogyrus* spp. from strictly Nearctic leuciscids (plus two *Dactylogyrus* spp. from catostomids) was also supported by the mapping of fish subfamilies into *Dactylogyrus* phylogeny by Šimková *et al.* [82], where cyprinids (with likely Asian origin) were shown as a potential ancestral host group for this parasite clade. Considering all the above, the “anchoratus” type of MCO (as defined by Pugachev *et al.* [66]) may represent the ancestral state for one of the main phylogenetic lineages of *Dactylogyrus* spp. parasitizing Nearctic cypriniforms (*i.e.*, species of clade A in the present study). This can be further supported by the fact that the MCO of the “anchoratus” type occurs in *D. yinwenyingae* Gussev, 1962, a species hitherto reported from nasal cavities of many cypriniforms (including pseudaspinins) from the Danube River up to the Amur River (see [66]) and water reservoirs of North America (=*Aplodiscus nasalis*; [72]). Finally, it is equally important to mention that the “nearctic” or “anchoratus” type of MCO can be observed in species of *Pellucidhaptor* Price & Mizelle, 1964. Species of this genus were found on the skin and in nasal cavities of catostomids, rarely leuciscids (Leuciscinae, Phoxininae, Pseudaspininae), from the Nearctic region (19 species) and the Palaearctic region (4 species) [41]. The haptoral configuration as well as the internal features of *Pellucidhaptor* spp. correspond to *Dactylogyru*s spp. with one haptoral bar (*e.g.*, *D. intermedius*, *D. vastator*, and *D. yinwenyingae*). The unclear morphological boundaries between these two genera together with the shared host spectrum may indicate an uncertain generic status for *Pellucidhaptor*. Thus, it would be interesting to investigate the phylogenetic position of *Pellucidhaptor* spp. within dactylogyrids parasitizing cypriniforms, especially their position to species of *Dactylogyrus*.

Unlike strictly Nearctic *Dactylogyrus* spp. (clade A), the Nearctic species forming a sister clade to European and North-West African *Dactylogyrus* spp. (clade B) exhibit variable morphologies of the MCOs, with the exception of *D. cheloideus* and *D. fimbratus* n. sp. from *Rhinichthys* spp. These two sister species share a similar morphology of both the MCO and haptoral structures, and, surprisingly, a similar MCO morphology with European *Dactylogyrus tincae* Gussev, 1968 and *Dactylogyrus triappendixis* Wierzbicka & Gronet, 1997 (for which molecular data are not available) from *Tinca tinca* (Linnaeus) (see [66]), an enigmatic species with a native Eurasian distribution [9, 28]. The European origin of Nearctic *Dactylogyrus* spp. forming clade B was previously suggested by Šimková *et al.* [82]. However, the basic MCO morphology of all four species corresponds to the “nearctic” (=anchoratus) type, the type characteristic of species of clade A, with likely Asian origin [82]. It is possible that the MCO of the “nearctic” type evolved convergently in *Dactylogyrus* spp. within both Nearctic lineages (*i.e.*, within clade A as well as in *D. cheloideus* and *D. fimbratus* n. sp. within clade B). However, there is also the possibility that the “nearctic” (=anchoratus) type is, in fact, the ancestral state of the MCO for *Dactylogyrus* spp. parasitizing European, West-African, and Nearctic cypriniforms, and that different MCO types in species of clade B developed after the divergence from a common ancestor (probably originating in the Oriental region) during the historical dispersion of Asian cyprinoids in Eurasia [8, 77].

Comparing the phylogenetic reconstruction and the morphology of the haptoral structures, *Dactylogyrus* species with the same configuration and similarly shaped haptoral structures tended to form monophyletic groups. This supports previous studies showing that haptoral structures reflect a phylogenetic signal and represent important tools for resolving monogenean phylogeny [4, 5, 48, 80, 86]. In the case of *Dactylogyrus* spp. parasitizing leuciscids of highly diversified Pogonichthyinae, two large morphological groups were recognized (corresponding to subclade A1 and subclade A2), each clearly defined by the shapes of the anchors and both bars. The third group sharing the same type of haptoral configuration consists of only two species, *D. cheloideus* and *D. fimbratus* n. sp., positioned within clade B. The remaining *Dactylogyrus* spp. from pogonichthyins have a different position (*D. aduncus* n. sp. *D. aviunguis*, and *D. lachneri* in clade A) or a poorly resolved position (*D. flagristylus* and *D. pectenatus* in clade B) in the phylogenetic tree, and possess different haptoral configurations. This seems to indicate that closely related *Dactylogyrus* spp. possess similar haptors not because they infect closely related host species (in this case, leuciscids of Pogonichthyinae) but because the same morphology of the haptor is shared from their common ancestor. It is generally accepted that the morphology of the haptoral structures determines specific gill microhabitat positions of monogenean species within a single host, and that coexisting species occupying the same niche should differ in their MCOs to strengthen reproductive barriers and thereby prevent hybridization through niche segregation (*e.g.* [56, 74, 79]). Alternatively, Vignon *et al.* [86] hypothesized that nonhomoplastic (*i.e.*, correlated with phylogeny) evolution of the haptoral sclerites may favor host-switch among closely related species in order to avoid hybridization. Similar patterns of host-parasite associations were observed in some cases of *Dactylogyrus* spp. investigated in the present study. Four species, *D. arcus*, *D. bulbus*, *D. cloutmani* n. sp., and *D. perlus*, co-occurred in *L. chrysocephalus isolepis* in Arkansas. The first three species share the same haptoral configuration as representatives of subclade A1, but they differ in the details of their MCOs (see Fig. 12). The fourth species, *D. perlus* (Fig. 4), clearly differs from the above three species by its distinctive morphology of the haptoral structures (*i.e.*, typical for species of subclade A2), and its presence on *L. chrysocephalus isolepis* likely represents host-switch. *Dactylogyrus* species forming subclade A2 show higher similarities in MCO morphology (see Fig. 13) compared to those of A1 – so much so that some of them appear to form complexes of species and their delimitation is controversial, as suggested for the *D. perlus* (=*banghami*) complex of species [16, 18, 32] reported from a variety of leuciscid hosts in North America [35]. Similarly, Cloutman *et al.* [20] raised the question of whether *D. crucis* and *D. lythruri* (*i.e.*, species with similar morphology to those in subclade A2) parasitizing the gills of seven and eight species of *Lythrurus*, respectively, display congeneric rather than strict host specificity or represent complexes of cryptic species. In the case of three *Dactylogyrus* species from *S. atromaculatus* (Plagopterinae) (group A3.1a) and two *Dactylogyrus* spp. from *H. nigricans* (Catostomidae) (group A3.1b), very similar haptoral and MCO morphologies for the respective groups were observed. The co-occurrence of *Dactylogyrus* spp. on one host species, as mentioned above, could indicate the same genetic/morphological underpinning of features leading to reproductive isolation and therefore parallel speciation among *Dactylogyrus* spp. from the respective hosts.

## Conclusion

In light of the above, further investigations based on precise morphological description, in particular detailed illustrations of the taxonomically important structures, and molecular characterization of the remaining species of *Dactylogyrus* should be performed to allow a more complete phylogenetic analysis of this diverse group of parasites. Many earlier taxonomic works on *Dactylogyrus* spp. in North America are based on schematic illustrations of the sclerotized parts of the attachment and reproductive organs, which probably led to poor differential diagnoses resulting in many errors in species identification, and even currently make species identification difficult. In this paper, seven new and 21 known species of *Dactylogyrus* previously molecularly determined are described and/or morphologically vouchered together with illustrations of the MCOs taken from the respective hologenophores. This study shows that a relatively simple accessory piece bifurcated into two unequal rami (right ramus is usually shorter and possesses lightly sclerotized pieces) should be considered the main synapomorphy of the phylogenetically strict Nearctic species of *Dactylogyrus*. Detailed illustration of the MCO is often the only tool for the differentiation of closely related species. *Dactylogyrus* spp. with the same configuration and similarly shaped haptoral structures tend to form monophyletic groups. The present results are a basic but important step to further ecological and evolutionary studies on this multi-continental group of monogenean parasites, including their host specificity and host-parasite co-evolutionary interactions.
